# New Triterpene Glycosides from the Far Eastern Starfish *Solaster pacificus* and Their Biological Activity

**DOI:** 10.3390/biom11030427

**Published:** 2021-03-14

**Authors:** Timofey V. Malyarenko, Alla A. Kicha, Anatoly I. Kalinovsky, Pavel S. Dmitrenok, Olesya S. Malyarenko, Alexandra S. Kuzmich, Valentin A. Stonik, Natalia V. Ivanchina

**Affiliations:** 1 G.B. Elyakov Pacific Institute of Bioorganic Chemistry, Far Eastern Branch of the Russian Academy of Sciences, Pr. 100-let Vladivostoku 159, 690022 Vladivostok, Russia; kicha@piboc.dvo.ru (A.A.K.); kaaniw@piboc.dvo.ru (A.I.K.); paveldmt@piboc.dvo.ru (P.S.D.); malyarenko.os@gmail.com (O.S.M.); assavina@mail.ru (A.S.K.); stonik@piboc.dvo.ru (V.A.S.); ivanchina@piboc.dvo.ru (N.V.I.); 2 Department of Bioorganic Chemistry and Biotechnology, School of Natural Sciences, Far Eastern Federal University, Sukhanova str. 8, 690000 Vladivostok, Russia

**Keywords:** triterpene glycosides, starfish, *Solaster pacificus*, biosynthesis, food markers, cytotoxic activity, colony formation

## Abstract

Three new triterpene glycosides, pacificusosides A–C (**1**–**3**), and three previously known triterpene glycosides, cucumariosides C_1_ (**4**), C_2_ (**5**), and A_10_ (**6**), were isolated from the alcoholic extract of the Far Eastern starfish *Solaster pacificus*. The structures of **1**–**3** were elucidated by extensive NMR and ESIMS techniques and chemical transformations. Compound **1** has a novel, unique structure, containing an aldehyde group of side chains in its triterpene aglycon. This structural fragment has not previously been found in the sea cucumber triterpene glycosides or starfish steroidal glycosides. Probably, pacificusoside A (**1**) is a product of the metabolism of the glycoside obtained through dietary means from a sea cucumber in the starfish. Another two new triterpene glycosides (**2**, **3**) have closely related characteristics to sea cucumber glycosides. The cytotoxicity of compounds **1**–**6** was tested against human embryonic kidney HEK 293 cells, colorectal carcinoma HT-29 cells, melanoma RPMI-7951 cells, and breast cancer MDA-MB-231 cells using MTS assay. Compounds **4**–**6** revealed the highest cytotoxic activity against the tested cell lines, while the other investigated compounds had moderate or slight cytotoxicity. The cytotoxic effects of **2**–**6** were reduced by cholesterol like the similar effects of the previously investigated individual triterpene glycosides. Compounds **3**, **4**, and **5** almost completely suppressed the colony formation of the HT-29, RPMI-7951, and MDA-MB-231 cells at a nontoxic concentration of 0.5 µM.

## 1. Introduction

The phylum Echinodermata includes five classes: Holothuroidea (sea cucumbers), Asteroidea (starfish), Echinoidea (sea urchins), Crinoidea (sea lilies), and Ophiuroidea (brittle stars and basket stars). These invertebrates are a rich source of a variety of low molecular metabolites with potent biological activities: triterpene glycosides, peptides, fatty acids, polar steroids and their glycosides, carotenoids, quinones, spinochromes pigments, and also sphingolipids and their derivatives [1].

Triterpene glycosides are considered to be characteristic secondary metabolites and chemotaxonomic markers of sea cucumbers. They are amphiphilic compounds consisting of lipophilic aglycon and a hydrophilic oligosaccharide chain [2]. Usually, these glycosides contain holostane type of aglycons having lanostane-3β-ol with (18,20)-lactone in the E-ring of the pentacyclic triterpene core. Some triterpene glycosides contain nonholostane aglycons [3]. As a rule, their carbohydrate chains include five or six monosaccharides such as D-xylose, D-quinovose, and D-glucose or their methylated or sulfated derivatives attached to C-3 of the aglycon. In the carbohydrate chains, the first sugar unit is always xylose, whereas 3-*O*-methyl-D-glucose and/or 3-*O*-methyl-D-xylose are predominantly the terminal monosaccharide units. These glycosides have been reported to exhibit a wide spectrum of biological activities such as cytotoxic [4,5], antifungal [6,7], bactericidal, hemolytic, antiviral, and antiparasitic properties [8]. Some triterpene glycosides were found to induce apoptosis and inhibit the growth of cancer cells [9], and show immunomodulatory properties [10].

At the same time, polar steroids and their glycosides are characteristic secondary metabolites of starfish (sea stars). The starfish steroidal glycosides may be divided into three major classes: glycosides of polyhydroxysteroids, cyclic glycosides, and asterosaponins [11,12,13,14,15,16,17,18,19]. Polyhydroxysteroidal glycosides from starfish have highly oxygenated steroidal aglycon. In most cases, monosaccharides of these compounds are represented by D-xylose and L-arabinose or their methylated or sulfated derivatives, forming monoside, bioside and/or sometimes trioside carbohydrate chains. Cyclic glycosides have several unusual structural features including a trisaccharide carbohydrate chain cyclized between C-3 and C-6 of the Δ^7^-3β,6β-dihydroxysteroidal aglycon as well as the presence of a glucuronic acid unit in the carbohydrate moiety. The majority of asterosaponins contain 3-*O*-sulfonated Δ^9(11)^-3β,6α-dihydroxysteroidal aglycon and carbohydrate chains, comprising usually five or six monosaccharide residues attached at the C-6 position of an aglycon.

It is worth noting that triterpene glycosides are not typical secondary metabolites of starfish. Earlier only two new triterpene glycosides, rollentosides A and B were isolated from the starfish *Asterias rollestoni* Bell, collected in the Yellow Sea near the shore of Jiangsu Province [20]. In addition, one previously known triterpene glycoside, holothurin A_2_ (= echinoside A), was isolated from the tropical starfish *Choriaster granulatus* [21]. In all other starfish triterpene glycosides were not found.

Herein, we report the results of our studies on triterpene glycosides from the ethanolic extract of the Far Eastern carnivorous starfish *Solaster pacificus* (order Valvatida, family Solasteridae) collected in the Sea of Okhotsk near Iturup Island. As a result, we have established the structures of three new triterpene glycosides, pacificusosides A–C (**1**–**3**), derived from the diet of this starfish. Moreover, we examined the cytotoxic activity of the individual compounds **1**–**6** or **1**–**6** in combination with cholesterol in human embryonic kidney, colorectal carcinoma, melanoma, and breast cancer cells. The effects of these compounds on the colony formation of the tested cancer cells were investigated using soft agar assay.

## 2. Materials and Methods

### 2.1. General Methods

Optical rotations were determined on a PerkinElmer 343 polarimeter (PerkinElmer, Waltham, MA, USA). UV spectra were recorded on a Shimadzu UV-1601 PC spectrophotometer (Shimadzu, Kioto, Japan). IR spectra were recorded using a Bruker Equinox 55 spectrophotometer (Bruker, Göttingen, Germany). The ^1^H and ^13^C NMR spectra were otained on Bruker Avance III 700 spectrometer (Bruker BioSpin, Bremen, Germany) at 700.13 and 176.04 MHz, respectively, chemical shifts were referenced to the corresponding residual solvent signals (*δ*_H_ 3.30/*δ*_C_ 49.0 for CD_3_OD). The HRESIMS spectra were recorded on a Bruker Impact II Q-TOF mass spectrometer (Bruker, Bremen, Germany); the samples were dissolved in MeOH (c 0.001 mg/mL). HPLC separations were carried out on an Agilent 1100 Series chromatograph (Agilent Technologies, Santa Clara, CA, USA) equipped with a differential refractometer; Diasfer-110-C18 (10 µm, 250 × 15 mm, Biochemmack, Moscow, Russia) and Discovery C_18_ (5 µm, 250 × 4 mm, Supelco, North Harrison, PA, USA) columns were used. Low-pressure liquid column chromatography was carried out with Polychrom-1 (powdered Teflon, 0.25−0.50 mm; Biolar, Olaine, Latvia), Si gel KSK (50–160 µm, Sorbpolimer, Krasnodar, Russia), and Florisil (60–100 µm, Sigma Aldrich, St. Louis, MO, USA). Sorbfil Si gel plates (4.5 × 6.0 cm, 5–17 µm, Sorbpolimer, Krasnodar, Russia) were used for thin-layer chromatography.

### 2.2. Animal Material

Specimens of *S. pacificus* Djakonov, 1938 (order Valvatida, family Solasteridae) were collected at a depth of 10 m–20 m by scuba diving in the Sea of Okhotsk near Iturup Island during the 42nd scientific cruise of the research vessel *Akademik Oparin* in August 2012. Species identification was carried out by Mr. B.B. Grebnev (G.B. Elyakov Pacific Institute of Bioorganic Chemistry of the FEB RAS, Vladivostok, Russia). A voucher specimen (no. 042–112) is on deposit at the marine specimen collection of the G.B. Elyakov Pacific Institute of Bioorganic Chemistry of the FEB RAS, Vladivostok, Russia.

### 2.3. Extraction and Isolation

The fresh animals (1.7 kg) were chopped and extracted twice with EtOH at 20 °C. The H_2_O/EtOH layer was evaporated, and the residue was dissolved in H_2_O (0.5 L). The H_2_O-soluble materials were passed through a Polychrom-1 column (6.5 × 21 cm), eluted with distilled H_2_O until a negative chloride ion reaction was obtained, and eluted with EtOH. The combined EtOH eluate was evaporated to give a brownish residue (48 g). These materials were chromatographed over a Si gel column (6 × 22 cm) using CHCl_3_/EtOH (stepwise gradient, 8:1 to 1:1), EtOH, and EtOH/H_2_O (4:1, 2:1, and 1:2) to yield fifteen fractions, 1–15, that were then analyzed by TLC on Si gel plates in the eluent system BuOH/EtOH/H_2_O (4:1:2). Fractions 4 (11.49 g) and 5 (22.79 g) were additionally chromatographed over a Florisil column (4.5 × 12 cm) using CHCl_3_/EtOH (stepwise gradient, 5:1 to 1:1) to yield three fractions, 41 (1.459 g), 42 (1.13 g), and 51 (652 mg). HPLC separation of fractions 41, 42, and 51 on a Diasfer-110-C18 column (10 μm, 250 × 15 mm, 2.5 mL/min) with EtOH/H_2_O (55:45) as an eluent system yielded pure 2 (31 mg, *t*_R_ 25.8 min), 3 (13.0 mg, *t*_R_ 91.5 min), 4 (89.0 mg, *t*_R_ 57.8 min), 5 (59 mg, *t*_R_ 71.2 min), and subfractions 41-3 (30 mg) and 42-1 (94.5 mg). HPLC separation of subfractions 41-3 and 42-1 on a Discovery C18 column (5 μm, 250 × 10 mm, 2.5 mL/min) with EtOH/H_2_O (50:50) as an eluent system yielded pure 1 (12.5 mg, *t*_R_ 6.6 min) and 6 (3.0 mg, *t*_R_ 16.3 min).

### 2.4. Spectral Data of New Compounds

*Pacificusoside A* (**1**), C_57_H_86_O_27_, white amorphous powder; [α]_D_^25^—27.7 (c 0.23, MeOH); UV (MeOH) *λ*_max_ 199, 224 nm; IR (KBr): *ν*_max_ = 3438, 2927, 1745, 1688, 1633, 1422, 1384, 1243, 1160, 1113, 1059, 894 cm^−1^; ^1^H and ^13^C NMR data are listed in Table 1 and Table 2; (+)ESIMS/MS of the ion [M+Na]^+^ at *m/z* 1225: 1093 [(M+Na)–C_5_H_8_O_4_]^+^; 947 [(M+Na)–C_6_H_10_O_4_–C_5_H_8_O_4_]^+^; 759 [(carbohydrate chain+Na)]^+^; 609 [(carbohydrate chain+Na)–C_5_H_8_O_4_–H_2_O]^+^; 477 [(carbohydrate chain+Na)–2×C_5_H_8_O_4_–H_2_O]^+^; 331 [(carbohydrate chain+Na)–2×C_5_H_8_O_4_–H_2_O–C_6_H_10_O_4_]^+^; 185 [C_6_H_10_O_5_+Na]^+^; 169 [C_6_H_10_O_4_+Na]^+^; (+)HRESIMS *m/z* 1225.5273 [M+Na]^+^ (calcd for [C_57_H_86_O_27_Na]^+^, 1225.5249); (–)ESIMS/MS of the ion [M–H]^−^ at *m/z* 1201: 1069 [(M–H)–C_5_H_8_O_4_]^−^; 923 [(M–H)–C_6_H_10_O_4_–C_5_H_8_O_4_]^−^; 761 [(M–H)–C_6_H_10_O_5_–C_6_H_10_O_4_–C_5_H_8_O_4_]^−^; 615 [(M–H)–C_6_H_10_O_5_–2×C_6_H_10_O_4_–C_5_H_8_O_4_]^−^; 483 [(M–H)–C_6_H_10_O_5_–2×C_6_H_10_O_4_–2×C_5_H_8_O_4_]^−^; (–)HRESIMS *m/z* 1201.5318 [M–H]^−^ (calcd for [C_57_H_85_O_27_]^−^, 1201.5284).

*Pacificusoside B* (**2**), C_53_H_82_O_24_, white amorphous powder; [α]_D_^25^—28.0 (c 0.1, MeOH); IR (KBr): *ν*_max_ = 3427, 2926, 2360; 1772, 1635, 1558, 1541, 1384, 1507, 1457, 1384, 1180, 1074 cm^−1^; ^1^H and ^13^C NMR data are listed in Table 1 and Table 2; (+)ESIMS/MS of the ion [M+Na]^+^ at *m/z* 1125: 993 [(M+Na)–C_5_H_8_O_4_]^+^; 979 [(M+Na)–C_6_H_10_O_4_]^+^; 847 [(M+Na)–C_6_H_10_O_4_–C_5_H_8_O_4_]^+^; 817 [(M+Na)–C_6_H_10_O_4_–C_6_H_10_O_5_]^+^; 759 [(carbohydrate chain+Na]^+^; 609 [(carbohydrate chain+Na)–C_5_H_8_O_4_–H_2_O]^+^; 477 [(carbohydrate chain+Na)–2×C_5_H_8_O_4_–H_2_O]^+^; 331 [(carbohydrate chain+Na)–2×C_5_H_8_O_4_–H_2_O–C_6_H_10_O_4_]^+^; 185 [C_6_H_10_O_5_+Na]^+^; 169 [C_6_H_10_O_4_+Na]^+^; (+)HRESIMS *m/z* 1125.5091 [M+Na]^+^ (calcd for [C_53_H_82_O_24_Na]^+^, 1125.5088); (–)ESIMS/MS of the ion [M–H]^−^ at *m/z* 1101: 969 [(M–H)–C_5_H_8_O_4_]^−^; 955 [(M–H)–C_6_H_10_O_4_]^−^; 823 [(M–H)–C_6_H_10_O_4_–C_5_H_8_O_4_]^−^; 793 [(M–H)–C_6_H_10_O_4_–C_6_H_10_O_5_]^−^; 661 [(M–H)–C_6_H_10_O_5_–C_6_H_10_O_4_–C_5_H_8_O_4_]^−^; 515 [(M–H)–C_6_H_10_O_5_–2×C_6_H_10_O_4_–C_5_H_8_O_4_]^−^; (–)HRESIMS *m/z* 1101.5119 [M–H]^−^ (calcd for [C_53_H_81_O_24_]^−^, 1101.5123).

*Pacificusoside C* (**3**), C_60_H_94_O_26_, white amorphous powder; [α]_D_^25^—9.0 (c 0.23, MeOH); IR (KBr): *ν*_max_ = 3427, 2927, 2854; 1745, 1635, 1457, 1384, 1238, 1180, 1065 cm^−1^; ^1^H and ^13^C NMR data are listed in Table 1 and Table 2; (+)ESIMS/MS of the ion [M+Na]^+^ at *m/z* 1253: 1121 [(M+Na)–C_5_H_8_O_4_]^+^; 759 [(carbohydrate chain+Na]^+^; 609 [(carbohydrate chain+Na)–C_5_H_8_O_4_–H_2_O]^+^; 477 [(carbohydrate chain+Na)–2×C_5_H_8_O_4_–H_2_O]^+^; 331 [(carbohydrate chain+Na)–2×C_5_H_8_O_4_–H_2_O–C_6_H_10_O_4_]^+^; 185 [C_6_H_10_O_5_+Na]^+^; 169 [C_6_H_10_O_4_+Na]^+^; (+)HRESIMS *m/z* 1253.5920 [M+Na]^+^ (calcd for [C_60_H_94_O_26_Na]^+^, 1253.5926); (–)ESIMS/MS of the ion [M–H]^−^ at *m/z* 1229: 1097 [(M–H)–C_5_H_8_O_4_]^−^; 1083 [(M–H)–C_6_H_10_O_4_]^−^; 951 [(M–H)–C_6_H_10_O_4_–C_5_H_8_O_4_]^−^; 921 [(M–H)–C_6_H_10_O_4_–C_6_H_10_O_5_]^−^; 789 [(M–H)–C_6_H_10_O_5_–C_6_H_10_O_4_–C_5_H_8_O_4_]^−^; 643 [(M–H)–C_6_H_10_O_5_–2×C_6_H_10_O_4_–C_5_H_8_O_4_]^−^; 511 [(M–H)–C_6_H_10_O_5_–2×C_6_H_10_O_4_–2×C_5_H_8_O_4_]^−^; (–)HRESIMS *m/z* 1229.5952 [M–H]^−^ (calcd for [C_60_H_93_O_26_]^−^, 1229.5961).

### 2.5. Acid Hydrolysis and Determination of Absolute Configurations of Monosaccharides

The acid hydrolysis of **1** (3.5 mg) was carried out in a solution of 2 M trifluoroacetic acid (TFA) (1 mL) in a sealed vial on a H_2_O bath at 100 °C for 2 h. The H_2_O layer was washed with CHCl_3_ (3 × 1.0 mL) and concentrated in vacuo. One drop of concentrated TFA and 0.5 mL of L-(–)-2-octanol (Sigma Aldrich) were added to the sugar mixture, and the sealed vial was heated on a glycerol bath at 130 °C for 6 h. The solution was evaporated in vacuo and treated with a mixture of pyridine/acetic anhydride (1:1, 0.6 mL) for 24 h at room temperature. The acetylated 2-octylglycosides were analyzed by GC using the corresponding authentic samples prepared by the same procedure. The following peaks were detected in the hydrolysate of **1**: D-quinovose (*t*_R_ 20.32, 20.55, 21.91, and 21.13 min), D-xylose (*t*_R_ 20.75, 20.91, and 21.19 min), D-glucose (*t*_R_ 24.24, 24.86, 25.10, and 25.38 min), and 3-OMe-D-xylose (*t*_R_ 19.33, 19.57, 19.84, and 20.00 min). The retention times of the authentic samples were as follows: D-quinovose (*t*_R_ 20.30, 20.50, 20.86, and 21.13 min), D-xylose (*t*_R_ 20.70, 20.89, and 21.15 min), D-glucose (*t*_R_ 24.23, 24.83, 25.06, and 25.37 min), 3-OMe-D-xylose (*t*_R_ 19.32, 19.57, 19.86, and 20.02 min), *L*-quinovose (*t*_R_ 20.12, 20.60, and 21.17 min), L-xylose (*t*_R_ 20.57, 21.03, and 21.34 min), L-glucose (*t*_R_ 24.39, 24.63, 24.83, and 25.06 min), and 3-OMe-L-xylose (*t*_R_ 18.92, 19.50, and 20.10 min).

### 2.6. Anticancer Activity

#### 2.6.1. Reagents

Phosphate buffered saline (PBS), L-glutamine, penicillin-streptomycin solution (10,000 U/mL, 10 µg/mL), dimethyl sulfoxide (DMSO), BuOH, 99.9% (pure p.a.), and cholesterol, 99.5% were from Sigma-Aldrich. MTS reagent (3-(4,5-dimethylthiazol-2-yl)-5-(3-carboxymethoxyphenyl)-2-(4-sulfophenyl)-2H-tetrazolium) was purchased from Promega (Madison, WI, USA). The Basal Medium Eagle (BME), Minimum Essential Medium Eagle (MEM), Dulbecco’s Modified Eagle’s Medium (DMEM), McCoy’s 5A Modified Medium (McCoy’s 5A), trypsin, fetal bovine serum (FBS), and agar were purchased from Thermo Fisher Scientific (Waltham, MA, USA). Organic solvents, inorganic acids, and salts were commercial products (Laverna, Moscow, Russia).

#### 2.6.2. Cell Lines and Culture Conditions

Human embryonic kidney HEK 293 cells (ATCC^®^ no. CRL-1573™), colorectal carcinoma HT-29 cells (ATCC^®^ no. HTB-38™), melanoma RPMI-7951 cells (ATCC^®^ no. HTB-66), and breast cancer MDA-MB-231 cells (ATCC^®^ HTB-26™) were obtained from the American Type Culture Collection (Manassas, VA, USA). HEK 293, HT-29, RPMI-7951, and MDA-MB-231cells were cultured in complete MEM/10% FBS, McCoy’s 5A/10% FBS, and DMEM/10% FBS, respectively, contained 1% of penicillin-streptomycin solution. The cell cultures were maintained at 37 °C in humidified atmosphere containing 5% CO_2_. Every 3–4 days cells were rinsed with PBS, detached from the tissue culture flask by 0.25% trypsin/0.05 M EDTA, and 10%–20% of the harvested cells were transferred to a new flask containing fresh culture media. After three passages the cells were used for the experiments.

#### 2.6.3. The Preparation of Solutions of Compounds

Compounds **1**–**6** were dissolved in sterile DMSO to prepare stock concentrations of 20 mM. Cells were treated with serially diluted compounds 1–6 (culture medium used as diluent) to give the intended final concentrations. The vehicle control is the cells treated with equivalent volume of DMSO (less than 0.5%) for all presented experiments.

Cholesterol (10 mg/mL) was dissolved in 99.5 % BuOH and added to compounds **1**–**6** to get their stock concentrations of 20 mM and incubated for 12 h at room temperature. Then samples were vacuum evaporated and dissolved in DMSO to their stock concentrations of 20 mM.

#### 2.6.4. Cell Viability Assay

Cells (1.0 × 10^4^) were seeded in 96-well plates (Jet Biofil, Guangzhou, China) and cultured in 200 µL of complete culture medium for 24 h at 37 °C in 5% CO_2_ incubator. The cell monolayer was treated with compounds 1–6 or 1–6 combined with cholesterol at concentrations of 5, 10, 20, and 40 µM in fresh appropriate culture medium for 24 h. Cells were also treated by cisplatin (positive control) at 1, 5, 10, 50, 100 µM for 24 and 48 h. Subsequently, the cells were incubated with 15 µL MTS reagent for 3 h, and the absorbance of each well was measured at 490/630 nm using Power Wave XS microplate reader (BioTek, Wynusky, VT, USA).

#### 2.6.5. Colony Formation Assay

Human cancer cells (2.4 × 10^4^/mL) were seeded into 6-well plate and treated with compounds **1**–**6** (0.1, 0.5, and 1 µM) or cisplatin (3 µM) in 1 mL of 0.3% BME agar containing 10% FBS, 2 mM L-glutamine, and 25 µg/mL gentamicin. The cultures were maintained in a 37 °C, 5% CO_2_ incubator for 14 days, and the cell colonies were scored using a Motic microscope AE 20 (XiangAn, Xiamen, China) and ImageJ software bundled with 64-bit Java 1.8.0_112 (NIH, Bethesda, MD, USA).

#### 2.6.6. Statistical Analysis

All assays were performed in at least three independent experiments. Results are expressed as the mean ± standard deviation (SD). Student’s t test was used to evaluate the data with the following significance levels: * *p* < 0.05, ** *p* < 0.01, *** *p* < 0.001.

## 3. Results

### 3.1. The Isolation and Structure Elucidation of Compounds **1**–**6** from S. pacificus

**Figure 1:**
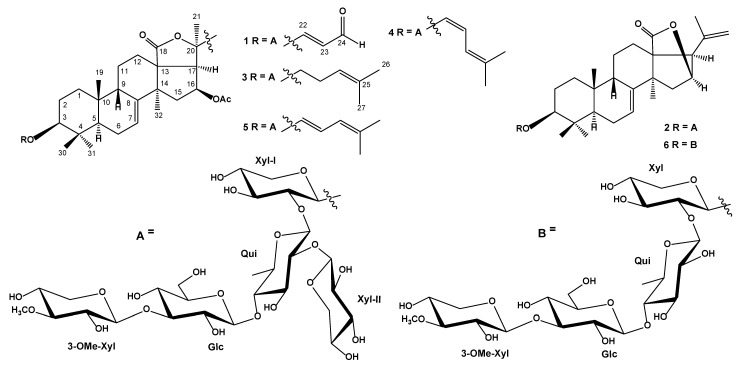
The structures of compounds **1**−**6** isolated from *S. pacificus.*

The concentrated ethanol extract of *S. pacificus* was subjected to sequential separation by chromatography on columns with Polichrom-1, Si gel, and Florisil followed by HPLC on semipreparative Diasfer-110-C18 and Discovery C_18_ columns to yield three new triterpene glycosides, named as pacificusosides A–C (**1**–**3**), and three previously known triterpene glycosides **4**–**6** (Figure 1). Compounds **4**–**6** were identified by comparison of their ^1^H and ^13^C NMR, and MS spectra with those reported for cucumariosides C_1_ (**4**), C_2_ (**5**), and A_10_ (**6**), triterpene glycosides from the holothurian *Eupentacta fraudatrix* [22,23].

**Figure 2:**
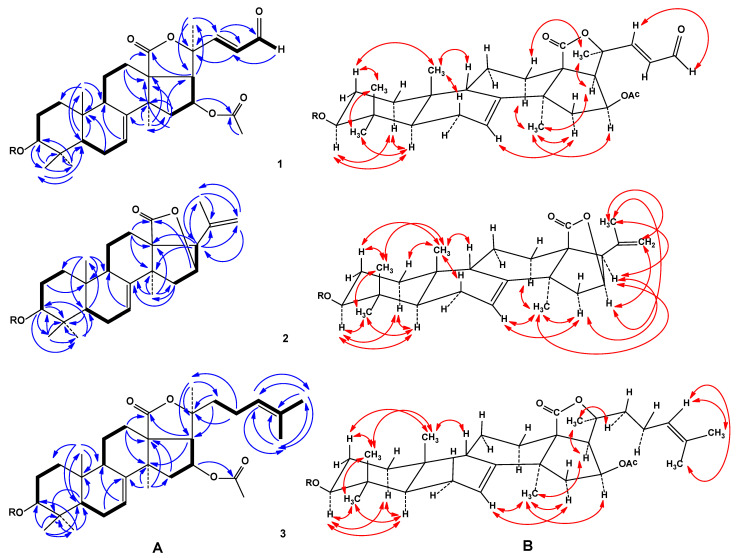
(**A**) ^1^H-^1^H COSY and key HMBC correlations for aglycon part of compounds **1**–**3**. (**B**) Key ROESY correlations for aglycon part of compounds **1**–**3**.

The molecular formula of **1** was determined to be C_57_H_86_O_27_ from the [M+Na]^+^ sodiated molecular ion peak at *m/z* 1225.5273 (calcd for [C_57_H_86_O_27_Na]^+^, 1225.5249) in the (+)HRESIMS and from the [M–H]^−^ ion peak at *m/z* 1201.5318 (calcd for [C_57_H_85_O_27_]^−^, 1201.5284) in the (–)HRESIMS (Figure S1). The IR spectrum of compound **1** showed the presence of hydroxy group (3438 cm^−1^), lactone carbonyl (1745 cm^−1^) and olefinic (1633 cm^−1^) groups, as well as one characteristic absorption band at *ν*_max_ = 1688 cm^−1^ of α,β-unsaturated aldehyde group (Figure S2). The ^1^H and ^13^C NMR spectroscopic data of the pentacyclic moiety of the aglycon of 1 showed the resonances of protons and carbons of four methyl groups (*δ*_H_ 1.15 s, 1.17 s, 1.24 s, 1.32 s; *δ*_C_ 32.3, 17.6, 24.1, 28.7), the 7(8)-double bond (*δ*_H_ 5.68 m; *δ*_C_ 121.1, 145.3), two oxygenated methine groups [*δ*_H_ 3.35 dd (*J* = 11.6, 4.1), 5.81 q (*J* = 8.3); *δ*_C_ 89.1, 73.6], one OAc-group (*δ*_H_ 1.86 s, *δ*_C_ 21.1, 169.7), and one lactone carbonyl (*δ*_C_ 178.5) (Table 1, Figures S4 and S5). The ^1^H-^1^H COSY and HSQC correlations attributable to triterpene nucleus revealed the corresponding sequences of protons from C-1 to C-3, C-5 to C-7, C-9 to C-12 through C-11, and C-15 to C-17 (Figure 2A, Figures S6 and S7). Key HMBC cross-peaks, such as H-3/C-30; H-5/C-10, C-19; H-6/C-10; H-7/C-9; H-12/C-13, C-14, C-18; H-15/C-13, C-14, C-17, C-32; H-16/CO(OAc); H-17/C-18, C-21; H_3_-19/C-1, C-5, C-9, C-10; H_3_-30/C-3, C-4, C-5, C-31; H_3_-31/C-3, C-4, C-5, C-30; H_3_-32/C-8, C-14, C-15; H_3_C(OAc)/CO(OAc) confirmed the overall structure of the pentacyclic triterpene moiety of 1 (Figure 2A, Figure S8). The key ROESY cross-peaks H-1α/H-3, H-5α; H-2β/H_3_-19, H_3_-30; H-3/H-5α; H-5α/H_3_-31; H-6β/H_3_-19; H-7/H-15α; H-9β/H_3_-19; H-12α/H_3_-21, H_3_-32; H-15α/H_3_-32; H-16α/H_3_-32; H-17α/H_3_-21, H_3_-32 showed the common 5α/9β/10β/13β,14α stereochemistry of the triterpene nucleus and 3β,16β-configurations of oxygenated substituents in **1** (Figure 2B, Figure S9).

Table 1 demonstrated the presence of one methyl group (*δ*_H_ 1.56 s; *δ*_C_ 28.9) and α,β-unsaturated aldehyde group [*δ*_H_ 7.28 d (*J* = 16.0), 6.46 dd (*J* = 16.0, 7.6), 9.78 d (7.6); *δ*_C_ 159.4, 127.6, 193.1] (Table 1, Figures S4 and S5). The existence of the α,β-unsaturated aldehyde group was confirmed by the UV spectrum (λ_max_ = 224 nm in MeOH) (Figure S3). The protons sequence from H-22 to H-24, correlated with the corresponding carbon atoms of the side chain of **1**, was assigned using the COSY and HSQC experiments (Table 1, Figures S6 and S7). The key HMBC correlations H_3_-21/C-17, C-20, C-22; H-22/C-20, C-23, C-24; H-23/C-20; and H-24/C-23 and ROESY correlation H-22/H-24 supported the total structure of the Δ^22^-24-aldo-25,26,27-trinorlanostane side chain (Figure 1). The *trans* configuration of 22(23)-double bond followed from *J*_22,23_ = 16.0 Hz. To the best of our knowledge, compound 1 has a novel type of triterpene aglycon, containing Δ^22^-25,26,27-trinorlanostane side chain with unique C-24 aldehyde group, never found before in the sea cucumber triterpene glycosides or starfish steroidal glycosides.

In addition to the above-mentioned signals, the ^1^H NMR spectrum of **1** exhibited five resonances in the deshielded region due to the anomeric protons of monosaccharide units at *δ*_H_ 4.84, 5.20, 4.94, 5.21, and 5.36 that correlated in the HSQC experiment with a carbon signals at *δ*_C_ 105.0, 103.0, 104.7, 106.0, and 105.8, respectively, as well as one resonance due to an *O*-methyl group at *δ*_H_ 3.85 that correlated in the HSQC experiment with a carbon signal at *δ*_C_ 60.4 (Table 2, Figures S4 and S5).

Along with mass spectral information, these data showed the presence of five monosaccharide residues in the oligosaccharide moiety of 1. The existence of 6-deoxy-sugar unit was supported by methyl doublet at *δ*_H_ 1.70. The coupling constants (7.0–8.0 Hz) of the anomeric protons were indicative of β-configurations of all glycosidic bonds. The chemical shifts and coupling constants of H-1′–H-6′ of the monosaccharide units were determined by irradiation of anomeric protons in the 1D TOCSY experiment. The ^1^H-^1^H COSY, HSQC, HMBC, and ROESY experiments led to the assignment of all the proton and carbon signals of the carbohydrate chain of 1 (Table 2, Figures S4–S9). The spectral data of the oligosaccharide moiety strictly coincided with those of terminal 3-*O*-methyl-β-D-xylopyranosyl and β-D-xylopyranosyl residues, and internal 3-*O*-substituted β-D-glucopyranosyl, 2,4-di-*O*-substituted β-D-quinovopyranosyl, and 2-*O*-substituted β-D-xylopyranosyl residues as in the earlier reported spectra of known cucumarioside C_1_ from *E. fraudatrix* [22].

On the basis of all the above-mentioned data, the structure of pacificusoside A (**1**) was determined as 3β-*O*-{3-*O*-methyl-β-D-xylopyranosyl-(1→3)-β-D-glucopyranosyl-(1→4)-[β-D-xylopyranosyl-(1→2)]-β-D-quinovopyranosyl-(1→2)-β-D-xylopyranosyl}-16β-acetoxy-25,26,27-trinorholosta-7,22-dien-24-al.

The molecular formula of **2** was determined to be C_53_H_82_O_24_ from the [M+Na]^+^ sodiated-molecular ion peak at *m/z* 1125.5091 (calcd for [C_53_H_82_O_24_Na]^+^, 1125.5088) in the (+)HRESIMS and from the [M–H]^−^ ion peak at *m/z* 1101.5119 (calcd for [C_53_H_81_O_24_]^−^, 1101.5123) in the (–)HRESIMS (Figure S10). The IR spectrum of compound **2** showed the presence of hydroxy group (3427 cm^−1^), lactone carbonyl (1772 cm^−1^), and olefinic (1635 cm^−1^) groups (Figure S11). The ^1^H and ^13^C NMR spectroscopic data belonging to the pentacyclic moiety of the aglycon of **2** showed the resonances of the protons and carbons of four methyl groups (*δ*_H_ 1.02 s, 1.12 s, 1.30 s, 1.35 s; *δ*_C_ 23.9, 17.3, 28.6, 33.9), a 7(8)-double bond [*δ*_H_ 5.64 brd (7.1); *δ*_C_ 122.6, 147.4), two oxygenated methine groups [*δ*_H_ 3.31 dd (*J* = 11.7, 3.8), 4.74 brd (*J* = 2.2); *δ*_C_ 88.9, 80.4], and one lactone carbonyl (*δ*_C_ 180.7) (Table 1, Figures S12 and S13). The proton and carbon signals of the OAc-group in the ^1^H and ^13^C NMR spectra of compound **2** were absent. The ^1^H-^1^H COSY and HSQC correlations attributable to triterpene nucleus revealed the corresponding sequences of protons from C-1 to C-3, C-5 to C-7, C-9 to C-12 through C-11, and C-15 to C-17 (Figure 2A, Figures S14 and S15). Key HMBC cross-peaks, such as H-2/C-10; H-3/C-4, C-30, C-31; H-5/C-4, C-19; H-7/C-9; H-12/C-13, C-14, C-18; H-15/C-8, C-32; H-16/C-18; H-17/C-13, C-14, C-20, C21, C-22; H_3_-19/C-1, C-5, C-9, C-10; H_3_-30/C-3, C-4, C-31; H_3_-31/C-4, C-5, C-30; H_3_-32/C-8, C-13, C-14, C-15 confirmed the overall structure of the pentacyclic triterpene moiety of 2 (Figure 2A, Figure S16). The key ROESY cross-peaks H-1α/H-3, H-5α; H-2β/H_3_-19, H_3_-30; H-3/H-5α, H_3_-31; H-5α/H_3_-31; H-6β/H_3_-19; H-7/H-15α, H_3_-32; H-9β/H_3_-19; H-12α/H_3_-32; H-15α/H-17α, H_3_-32; H-17α/H_3_-32 showed the common 5α/9β/10β/13β,14α stereochemistry of the triterpene nucleus and 3β,16β-configurations of oxygenated substituents in 2 (Figure 2B, Figure S17). The proton and carbon signals of the aglycon side chain of **2** demonstrated the presence of only one methyl group (*δ*_H_ 1.75 s; *δ*_C_ 23.0) and one 20,22-double bond (*δ*_H_ 5.06 s, 4.98 s; *δ*_C_ 139.9, 113.9). The key HMBC correlations H_3_-21/C-17, C-20, C-22 and H-22/C-17, C-21 and the key ROESY correlations H_3_-21/H-16, H-17, H-22; and H_2_-22/H-16 supported the total structure of the side chain (Figure 2A,B). The NMR spectroscopic data of aglycon part of 2 were coincident with those for known cucumariosides A_10_ and I_4_ from the sea cucumber *E. fraudatrix* with 23,24,25,26,27-pentanorlanosta-7,20(22)-diene-18(16)-lactone-3β-ol aglycon [23,24], but the carbohydrate chain was different.

On the basis of extensive 2D NMR and MS analysis of glycosides **1**–**3**, we have suggested that oligosaccharide moiety of **1** are identical to those of glycosides **2** and **3**. On the basis of all the above-mentioned data, the structure of pacificusoside B (**2**) was elucidated to be 3β-*O*-{3-*O*-methyl-β-D-xylopyranosyl-(1→3)-β-D-glucopyranosyl-(1→4)-[β-D-xylopyranosyl-(1→2)]-β-D-quinovopyranosyl-(1→2)-β-D-xylopyranosyl}-23,24,25,26,27-pentanorlanosta-7,20(22)-diene-18(16)-lactone.

The molecular formula of **3** was determined to be C_60_H_94_O_26_ from the [M+Na]^+^ sodiated-molecular ion peak at *m/z* 1253.5920 (calcd for [C_60_H_94_O_26_Na]^+^, 1253.5926) in the (+)HRESIMS and from the [M–H]^−^ ion peak at *m/z* 1229.5952 (calcd for [C_60_H_93_O_26_]^−^, 1229.5961) in the (–)HRESIMS (Figure S18). The IR spectrum of compound 3 showed the presence of hydroxy (3427 cm^−1^), lactone carbonyl (1745 cm^−1^), and olefinic (1635 cm^−1^) groups (Figure S19). The thorough comparison of the ^1^H and ^13^C NMR data of compound **3** with those of **1** (Figures S4, S5, S20, and S21) showed that they differed from each other only in the signals of their side chains (Table 1). The proton and carbon signals of aglycon side chain of 3 demonstrated the presence of three methyl groups (*δ*_H_ 1.53 s, 1.59 s, 1.65 s; *δ*_C_ 28.5, 17.9, 25.7) and one double bond (*δ*_H_ 5.12 m; *δ*_C_ 124.4, 132.1). The protons sequence from H-22 to H-27, correlated with the corresponding carbon atoms of the side chain of 3, was assigned using the COSY and HSQC experiments (Table 1, Figures S22 and S23). The key HMBC correlations H_3_-21/C-17, C-20, C-22; H-22/C-17, C-20; H-23/C-20, C-21; H-24/C-26, C-27; H_3_-26/C-24, C-25, C-27, and H_3_-27/C-24, C-25, C-26 and key ROESY correlations H_3_-21/H-17, H-22 and H-24/H_3_-27, H_3_-26 supported the total structure of the Δ^24^-lanostane side chain (Figure 1). Thus, aglycon of glycoside 3 was identical to aglycon of known cucumarioside A_1_ from *E. fraudatrix* [25]. Therefore, the structure of pacificusoside C (3) was established as 3β-*O*-{3-*O*-methyl-β-D-xylopyranosyl-(1→3)-β-D-glucopyranosyl-(1→4)-[β-D-xylopyranosyl-(1→2)]-β-D-quinovopyranosyl-(1→2)-β-D-xylopyranosyl}-16β-acetoxyholosta-7,24-diene.

### 3.2. In Vitro Anticancer Activity of Compounds **1**–**6**

The cytotoxicity of investigated compounds **1**–**6** was tested against human embryonic kidney HEK 293 cells, colorectal carcinoma HT-29 cells, melanoma RPMI-7951 cells, and breast cancer MDA-MB-231 cells using MTS assay. The results of the experiment are expressed in IC_50_ values (Table 3). The compounds 4–6 reveal high cytotoxic activity against the tested cell lines (Table 3, column a). Compound 3 has comparable high cytotoxic activity against HEK 293 (IC_50_ = 6.7 ± 0.2), HT-29 (IC_50_ = 6.2 ± 0.3), and MDA-MB-231 (IC_50_ = 6.4 ± 0.4) cells and moderate effect against RPMI-7951 cells (IC_50_ = 23.7 ± 0.4). Compound 2 inhibits cell viability of all tested cell lines with IC_50_ value more than 20 µM (Table 3, column a). In contrast, compound 1 was nontoxic against all types of cells at concentrations range up to 40 µM (Table 3, column a). Cisplatin, a well-known chemotherapeutic drug, was used as the reference cytotoxic drug (positive control). IC_50_ of cisplatin against HEK 293, HT-29, RPMI-7951, and MDA-MB-231 cells was 64.6 µM, 95.4 µM, 48.4 µM, and 7.8 µM, respectively, after 48 h of treatment (data not shown).

Such potent cytotoxicity of compounds **3**–**6** can be explained by the fact that the studied triterpene glycosides are capable of interacting with cholesterol of cellular membranes, which leads to cell death as a result of disruption or loss of the integrity of the cell membrane. Since cholesterol is known for its function of stabilizing the cell membrane, the loss of cholesterol itself or the loss of its ability to perform this function is undoubtedly a reason of the observed instability of the cellular membranes and membranolysis of the target cells [26,27,28]. Since the triterpene saponins are able to complex cholesterol [29], we analyzed the influence of triterpene glycosides **2**–**6** combined with cholesterol on the cell viability. It was found that the cytotoxic activity of **2**–**6** combined with cholesterol was greatly reduced compared with activity of the investigated individual triterpene glycosides (Table 3, column b). The IC_50_ value of compounds **3**–**5** was increased by approximately 3 times, and compound 6—by 1.5 times (Table 3, column b). Obtained data suggested that mechanism of cytotoxic effect of triterpene glycosides **2**–**6** may be related to binding of membrane cholesterol resulting in membranolysis and cell death. Several works confirmed that triterpene glycosides from sea cucumbers possess potent membranolytic activity [30,31].

It was demonstrated that the investigated compounds **1**–**6** (at concentrations 5, 10, 20, and 40 µM) did not show selective cytotoxicity on cancer cells, because the viability of normal cells was also suppressed. That is why we investigated the effect of compounds 1–6 on colony formation at the nontoxic concentrations of 0.1, 0.5, and 1 µM, at which they did not influence the viability of normal HEK 293 cells (data not shown).

**Figure 3:**
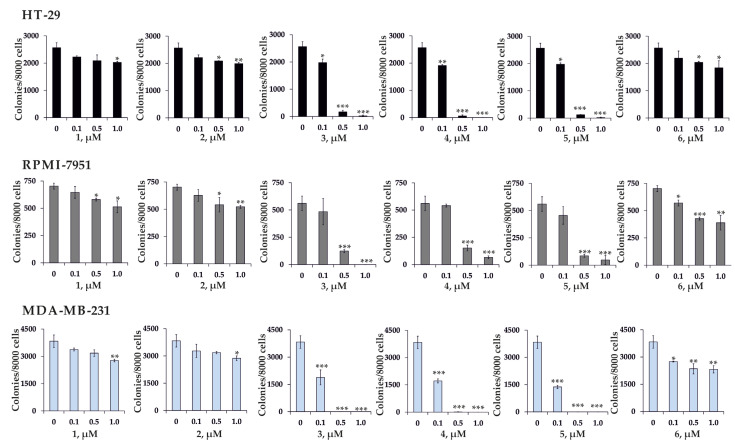
Effect of compounds **1**–**6** on colony formation of human colorectal carcinoma HT-29, melanoma RPMI-7951, and breast cancer MDA-MB-231 cell lines. HT-29, RPMI-7951, and MDA-MB-231 cells (2.4 × 10^4^ /mL) treated with/without investigated 1–6 (0.1, 0.5, and 1 µM) and subjected into a soft agar. The culture was maintained at 37 °C in a 5% CO_2_ atmosphere for 2 weeks. The colonies were counted under a microscope with the aid of the ImageJ software program (*n* = 6 for control and each compound, n—quantity of photos). The asterisks (* *p* < 0.05, ** *p* < 0.01, *** *p* < 0.001) indicate a significant decrease in colony formation in cells treated with compounds compared with the nontreated cells (control).

Compounds **1**, **2**, and **6** at a concentration of 1 µM possess moderate inhibitory activity on the model of the colony formation of the cancer cells, namely, 1, 2, and 6 decreased number of colonies of HT-29 by 21, 22, and 28%, respectively; RPMI-7951—by 27%, 26%, and 45%, respectively; MDA-MB-231—by 28, 25, and 40%, respectively (Figure 3). Compounds 3, 4, and 5 almost completely suppressed colony growth of HT-29, RPMI-7951, and MDA-MB-231 cells at concentrations of 0.5 µM and 1 µM (Figure 3). To the best of our knowledge this is the first evidence that triterpene glycosides at nontoxic concentrations significantly inhibited the colony formation of human cancer cells.

## 4. Conclusions

The isolation of a series of new triterpene glycosides from starfish is a rare case. Previously, triterpene glycosides, characteristic metabolites of sea cucumbers, were isolated only from two species of starfish: *A. rollestoni* Bell and *C. granulatus* [20,21]. Pacificusosides A–C (**1**–**3**), new glycosides from the starfish *S. pacificus* have a great structural similarity with the triterpene glycosides earlier obtained from the sea cucumber *E. fraudatrix*. In particular, the isolated glycosides 1, 3 contain the holostane type of aglycons with 16β-acetoxy group and 7(8)-double bond and glycoside 2 contains the rare nonholostane type aglycon, 23,24,25,26,27-pentanorlanosta-7,20(22)-diene-18(16)-lactone-3β-ol.

The oligoglycoside chains of 1–3 have 3-*O*-methyl-D-xylose as a terminal monosaccharide unit. This monosaccharide residue was previously found only in the sea cucumber *E. fraudatrix*. It was previously suggested that this structural peculiarity is a chemotaxonomic marker of *E. fraudatrix* [32]. The finding of 3-*O*-methyl-D-xylose unit in pacificusosides A–C (1–3) from the starfish shows that these glycosides are food markers. Probably, these compounds are partly metabolized in the starfish glycosides from the sea cucumber, belonging to the genus *Eupentacta* and obtained with diet. The studied starfish has the synonymized name: *Solaster endeca pacificus* (Djakonov, 1938). It was reported that in the Atlantic Ocean *S. endeca pacificus* feeds on other starfish and bivalve mollusks, but in the Pacific, its diet is mainly sea cucumbers and other invertebrates [33]. Without doubt, the starfish *S. pacificus* feeds on sea cucumbers, as was reported earlier.

**Figure 4:**
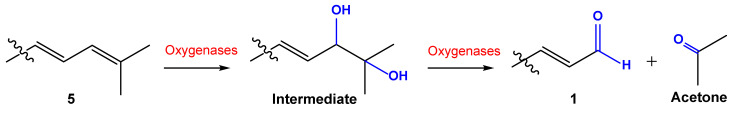
Hypothetic scheme of the biosynthesis of aglycon side chain of compound **1**.

At the same time, glycoside 1 has a novel unique type of side chain, containing the aldehyde group in the triterpene aglycon. This structural fragment was not previously found in the sea cucumber triterpene glycosides or sea star steroidal glycosides. Obviously, pacificusoside A is a metabolite, formed by the transformation of a toxic glycoside from a diet in the starfish. It is of particular interest that modified glycoside (1) is significantly less cytotoxic when compared with intact cucumariosides from *E. fraudatrix*. We assumed that the formation of an unusual glycoside, pacificusoside A (1), from related glycosides of the starfish is carried out in two stages. At the first stage, cucumarioside C_2_ (5) from the sea cucumber *E. fraudatrix*, most probably the biosynthetic precursor of pacificusoside A (1), is oxidized by oxygenases similar to P_450_ cytochromes. At the second stage, the shortening of the Δ^22,24^-lanostane side chain of compound 5 occurs in the starfish *S. pacificus* with the formation of the Δ^22^-24-aldo-25,26,27-trinorlanostane side chain in glycoside 1 (Figure 4). Presumably, the cleavage of part of the side chain occurs under the action of specific oxygenases, similarly to the formation of pregnenolone from cholesterol under the action of cytochrome P450_scc_ in mammals during the synthesis of progesterone. The existence of such oxygenases in sea stars can be confirmed by the detection of (24*R*)-5α-cholestane-3β,4β,6β,8,15β,24,25-heptaol, which has hydroxy groups at C-24 and C-25 in cholestane side chain [34] as in the intermediate on Figure 4.

We believe that the formation of similar compounds with an aldehyde group in the side chain of glycosides 3 and 4 is also possible, but requires more time after feeding. Moreover, the slow metabolism of the toxic triterpene glycosides in starfish allows them to use the accumulated dietary ichthyotoxic compounds to defend themselves against predators.

Thus, the oxidation with a subsequent decrease in the number of carbon atoms in the side chain of the triterpene glycosides partly led to the decrease in the cytotoxicity of the modified compounds. Indeed, based on the obtained data, one can see a pattern between the structure and biological activity of the isolated compounds. The highest cytotoxicity against all types of cancer and normal cells, as well as the highest inhibitory effect on colony formation of cancer cells, were demonstrated by glycosides 3–5, which have Δ^24^-lanostane and *cis*- and *trans*-Δ^22,24^-lanostane side chains in the triterpene aglycon, respectively. At the same time, the IC_50_ of pacificusoside A (1) on all cell types was more than 40 μM, and this compound also slightly inhibited the colony formation of all types of cancer cells in a soft agar assay. According to the data of the colony formation assay, the glycosides 2 and 6, which have a triterpene aglycon with a rearranged 18(16)-lactone, are less active than glycosides 3–5, which have a holostane triterpene aglycon with an 18(20)-lactone.

Finally, we once more confirmed the hypothesis that triterpene glycosides are membranolytic and exert their cytotoxic effect by binding the components of the plasma membrane of target cells. In fact, when cholesterol was combined with the compounds **1**–**6**, the cytotoxicity of all glycosides decreased markedly. This is likely due to the fact that some of the molecules of triterpene glycosides **1**–**6** bind the cholesterol added to these compounds.

On the other hand, glycosides **3**–**5**, which showed significant or complete inhibition the colony formation of cancer cells at nontoxic concentrations, can be further studied as potential anticancer agents.

## Figures and Tables

**Table 1:** ^1^H and ^13^C NMR data of aglycon part of compounds **1**–**3** (35 °C, C_5_D_5_N, *J* in Hz) ^a^.

Position	1		2		3	
	*δ*_H_	*δ*_C_	*δ*_H_	*δ*_C_	*δ*_H_	*δ*_C_
1	1.49 m	36.3	1.48 m	35.9	1.48 m	36.4
2	2.21 m 1.96 m	27.2	2.16 m 1.89 m	26.9	2.19 m 1.94 m	27.3
3	3.35 dd (11.6, 4.1)	89.1	3.31 dd (11.7, 3.8)	88.9	3.34 dd (11.5, 3.9)	89.3
4	-	39.7	-	39.4	-	39.8
5	1.06 m	48.1	1.00 dd (11.9, 3.6)	47.5	1.06 m	48.3
6	2.08 m	23.4	2.07 m 1.97 m	23.3	2.06 m 1.93 m	23.6
7	5.68 m	121.1	5.64 brd (7.1)	122.6	5.68 brs	120.7
8	-	145.3	-	147.4	-	145.9
9	3.44 brd (14.2)	47.4	3.00 brd (14.6)	46.4	3.48 brd (13.5)	47.4
10	-	35.8	-	35.5	-	35.8
11	1.88 m 1.60 m	22.5	2.00 m 1.47 m	21.7	1.81 m 1.55 m	22.8
12	2.15 m 2.02 m	30.7	2.37 m 1.88 m	20.0	2.16 m 2.00 m	31.6
13	-	59.1	-	56.7	-	59.5
14	-	48.2	-	46.0	-	47.7
15	2.58 dd (12.5, 7.2) 1.70 m	43.3	2.16 d (13.3) 1.97 dd (13.3, 2.2)	43.8	2.63 dd (12.4, 7.4) 1.75 m	43.9
16	5.81 q (8.3)	73.6	4.74 brd (2.2)	80.4	5.96 q (8.7)	75.2
17	3.01 d (8.3)	56.8	2.94 s	59.1	2.66 d (9.5)	54.8
18	-	178.5	-	180.7	-	179.7
19	1.24 s	24.1	1.02 s	23.9	1.23 s	24.2
20	-	82.4	-	139.9	-	85.2
21	1.56 s	28.9	1.75 s	23.0	1.53 s	28.5
22	7.28 d (16.0)	159.4	5.06 s 4.98 s	113.9	2.57 td (12.0, 4.4) 1.93 m	38.9
23	6.46 dd (16.0, 7.6)	127.6	-	-	2.07 m2.01 m	23.6
24	9.78 d (7.6)	193.1	-	-	5.12 m	124.4
25	-	-	-	-	-	132.1
26	-	-	-	-	1.59 s	17.9
27	-	-	-	-	1.65 s	25.7
30	1.17 s	17.6	1.12 s	17.3	1.15 s	17.7
31	1.32 s	28.7	1.30 s	28.6	1.31 s	29.0
32	1.15 s	32.3	1.35 s	33.9	1.17 s	32.4
CO	-	169.7	-	-	-	170.0
CH_3_-CO	1.86 s	21.1	-	-	2.01 s	21.2

^a^ Assignments from 700 MHz ^1^H-^1^H COSY, HSQC, HMBC (8 Hz), and ROESY (270 msec) data.

**Table 2:** ^1^H, ^13^C, HMBC, and ROESY NMR data of oligosaccharide chains of 1–3 (35 °C, C_5_D_5_N, *J* in Hz) ^a^.

Position	1–3			
	*δ*_H_	*δ*_C_	HMBC	ROESY
	*Xyl-I*			
1	4.84 d (7.1)	105.0	C3-Agl; C5-Xyl-I	H3, H30-Agl; H3, H5-Xyl-I
2	3.91 dd (8.7, 7.1)	83.2	C1, C3-Xyl-I; C1-Qui	H4-Xyl-I
3	4.19 t (8.7)	77.8	C2, C4-Xyl-I	H1-Xyl-I
4	4.11 m	70.2	C3-Xyl-I	H2-Xyl-I
5	4.32 dd (11.6, 5.4) 3.69 dd (11.6, 9.3)	66.5	C1, C3-Xyl-I C1, C3-Xyl-I	H1-Xyl-I
	*Qui*			
1	5.20 d (7.3)	103.0	C2-Xyl-I	H3, H5-Qui; H2-Xyl-I
2	4.11 m	82.7	C1, C3-Qui; C1-Xyl-II	
3	4.09 m	75.6	C2, C4-Qui	H1-Qui
4	3.61 m	86.5	C3, C5, C6-Qui; C1-Glc	H1-Glc; H6-Qui
5	3.67 m	71.0	C4-Qui	H1-Qui
6	1.70 d (6.5)	17.9	C4, C5-Qui	H4-Qui
	*Glc*			
1	4.94 d (7.9)	104.7	C4-Qui	H4-Qui; H3, H5-Glc
2	4.00 m	73.8	C1, C3-Glc	
3	4.21 t (9.0)	87.1	C2, C4-Glc	H1-3-OMe-Xyl; H1, H5-Glc
4	4.05 t (9.0)	69.3	C3, C5, C6-Glc	
5	3.99 m	77.8		H1, H3-Glc
6	4.47 dd (11.5, 2.6) 4.20 m	61.7		
	*3-OMe-Xyl*			
1	5.21 d (7.6)	106.0	C3-Glc; C5-3-OMe-Xyl	H3, H5-3-OMeXyl; H3-Glc
2	3.94 t (8.4)	74.6	C1, C3-3-OMe-Xyl	H4-3-OMe-Xyl
3	3.60 t (8.8)	87.7	C2, C4-3-OMe-Xyl; OMe	H1-3-OMe-Xyl; OMe
4	4.08 m	69.9		H2-3-OMe-Xyl
5	4.21 dd (11.3, 5.5) 3.63 t (11.3)	67.0	C1, C3-3-OMe-Xyl C1, C3, C4-3-OMe-Xyl	H1-3-OMe-Xyl
3-OMe	3.85 s	60.4	C3-3-OMe-Xyl	H3-3-OMe-Xyl
	*Xyl-II*			
1	5.36 d (7.0)	105.8	C2-Qui; C5-Xyl-II	H3, H5-Xyl-II; H2-Qui
2	4.04 m	75.5	C1, C3-Xyl-II	
3	4.10 t (8.1)	77.0	C2, C4-Xyl-II	H1-Xyl-II
4	4.12 m	70.4	C3-Xyl-II	
5	4.33 dd (11.7, 4.6) 3.65 dd (11.7, 9.6)	66.9	C1, C3, C4-Xyl-II C1, C4, C5-Xyl-II	H1-Xyl-II

^a^ Assignments from 700 MHz ^1^H-^1^H COSY, HSQC, HMBC (8 Hz), and ROESY (270 msec) data.

**Table 3:** Cytotoxic activities of compounds **1**–**6** and their mixtures with cholesterol. Values are indicated as mean ± standard deviation.

Compound	Inhibiting Concentration, (IC_50_), µM							
	HEK 293		HT-29		RPMI-7951		MDA-MB-231	
	a	b	a	b	a	b	a	b
**1**	>40	n.d.	>40	n.d.	>40	n.d.	>40	n.d.
**2**	28.6 ± 2.1	>40	26.2 ± 2.5	>40	20.4 ± 1.6	>40	21.5 ± 0.5	>40
**3**	6.7 ± 0.2	24.2 ± 4.0	6.2 ± 0.3	19.4 ± 3.3	23.7 ± 0.4	>40	6.4 ± 0.4	20.0 ± 4.7
**4**	5.5 ± 0.3	17.3 ± 0.8	5.3 ± 0.2	14.3 ± 3.0	5.8 ± 1.2	16.4 ± 3.1	3.6 ± 0.2	12.8 ± 0.7
**5**	7.9 ± 0.9	19.5 ± 1.2	5.5 ± 0.6	15.9 ± 0.26	5.9 ± 0.9	17.0 ± 2.8	4.4 ± 0.09	12.9 ± 2.5
**6**	6.3 ± 0.6	13.2 ± 0.5	7.8 ± 0.7	10.9 ± 0.05	5.8 ± 2.6	11.4 ± 0.8	5.5 ± 0.1	10.5 ± 1.8

IC_50_, the concentration of compounds that caused a 50% reduction in cell viability of tested normal and cancer cells. a IC_50_ of individual compounds **1**–**6** for 24 h. b IC_50_ of **1**–**6** combined with cholesterol for 24 h. n.d.—not determined.

## Data Availability

The data presented in this study are available on request from the corresponding authors.

## Supplementary Materials

The following are available online at https://www.mdpi.com/2218-273X/11/3/427/s1, Figure S1: (–)HRESIMS spectrum of pacificusoside A (1); Figure S2: IR spectrum of pacificusoside A (1) in KBr; Figure S3: UV spectrum of pacificusoside A (1) in MeOH; Figure S4: ^1^H NMR spectrum of pacificusoside A (1) in C_5_D_5_N; Figure S5: ^13^C NMR spectrum of pacificusoside A (1) in C_5_D_5_N; Figure S6: ^1^H-^1^H COSY spectrum of pacificusoside A (1) in C_5_D_5_N; Figure S7: HSQC spectrum of pacificusoside A (1) in C_5_D_5_N; Figure S8: HMBC spectrum of pacificusoside A (1) in C_5_D_5_N; Figure S9: ROESY spectrum of pacificusoside A (1) in C_5_D_5_N; Figure S10: (–)HRESIMS spectrum of pacificusoside B (2); Figure S11: IR spectrum of pacificusoside B (2) in KBr; Figure S12: ^1^H NMR spectrum of pacificusoside B (2) in C_5_D_5_N; Figure S13: ^13^C NMR spectrum of pacificusoside B (2) in C_5_D_5_N; Figure S14: ^1^H-^1^H-COSY spectrum of pacificusoside B (2) in C_5_D_5_N; Figure S15: HSQC spectrum of pacificusoside B (2) in C_5_D_5_N; Figure S16: HMBC spectrum of pacificusoside B (2) in C_5_D_5_N; Figure S17: ROESY spectrum of pacificusoside B (2) in C_5_D_5_N; Figure S18: (–)HRESIMS spectrum of pacificusoside C (3); Figure S19: IR spectrum of pacificusoside C (3) in KBr, Figure S20: ^1^H NMR spectrum of pacificusoside C (3) in C_5_D_5_N; Figure S21: ^13^C NMR spectrum of pacificusoside C (3) in C_5_D_5_N; Figure S22: ^1^H-^1^H COSY spectrum of pacificusoside C (3) in C_5_D_5_N; Figure S23: HSQC spectrum of pacificusoside C; (3) in C_5_D_5_N; Figure S24: HMBC spectrum of pacificusoside C; (3) in C_5_D_5_N; Figure S25: ROESY spectrum of pacificusoside C; (3) in C_5_D_5_N.
