# Supplementary material for: New Triterpene Glycosides from the Far Eastern Starfish Solaster pacificus and Their Biological Activity
Source: Biomolecules. 2021 Mar 14;11(3):427. doi: 10.3390/biom11030427 (PMC8001898; doi:10.3390/biom11030427)

Supplementary Materials

New Triterpene Glycosides from the Far Eastern Starfish *Solaster pacificus* and Their Biological Activity

Timofey V. Malyarenko[1,2*], Alla A. Kicha[1], Anatoly I. Kalinovsky[1], Pavel S. Dmitrenok[1], Olesya S. Malyarenko[1], Alexandra S. Kuzmich[1], Valentin A. Stonik[1,2], and Natalia V. Ivanchina[1]

[1] G.B. Elyakov Pacific Institute of Bioorganic Chemistry, Far Eastern Branch of the Russian Academy of Sciences, Pr. 100-let Vladivostoku 159, 690022 Vladivostok, Russia; kicha@piboc.dvo.ru (A.A.K.); kaaniw@piboc.dvo.ru (A.I.K.); paveldmt@piboc.dvo.ru (P.S.D.); malyarenko.os@gmail.com (O.S.M.); assavina@mail.ru (A.S.K.); stonik@piboc.dvo.ru (V.A.S.); ivanchina@piboc.dvo.ru (N.V.I.).

[2] Far Eastern Federal University, Sukhanova str. 8, 690000 Vladivostok, Russia

List

Figure S1. (–)HRESIMS spectrum of pacificusoside A (**1**).

Figure S2. IR spectrum of pacificusoside A (**1**) in KBr.

Figure S3. UV spectrum of pacificusoside A (**1**) in MeOH.

Figure S4. ^{1}H NMR spectrum of pacificusoside A (**1**) in C_5D_5N.

Figure S5. ^{13}C NMR spectrum of pacificusoside A (**1**) in C_5D_5N.

Figure S6. ^{1}H-^{1}H COSY spectrum of pacificusoside A (**1**) in C_5D_5N.

Figure S7. HSQC spectrum of pacificusoside A (**1**) in C_5D_5N.

Figure S8. HMBC spectrum of pacificusoside A (**1**) in C_5D_5N.

Figure S9. ROESY spectrum of pacificusoside A (**1**) in C_5D_5N.

Figure S10. (–)HRESIMS spectrum of pacificusoside B (**2**).

Figure S11. IR spectrum of pacificusoside B (**2**) in KBr.

Figure S12. ^{1}H-NMR spectrum of pacificusoside B (**2**) in C_5D_5N.

Figure S13. ^{13}C-NMR spectrum of pacificusoside B (**2**) in C_5D_5N.

Figure S14. ^{1}H-^{1}H-COSY spectrum of pacificusoside B (**2**) in C_5D_5N.

Figure S15. HSQC spectrum of pacificusoside B (**2**) in C_5D_5N.

Figure S16. HMBC spectrum of pacificusoside B (**2**) in C_5D_5N.

Figure S17. ROESY spectrum of pacificusoside B (**2**) in C_5D_5N.

Figure S18. (–)HRESIMS spectrum of pacificusoside C (**3**).

Figure S19. IR spectrum of pacificusoside C (**3**) in KBr.

Figure S20. ^{1}H-NMR spectrum of pacificusoside C (**3**) in C_5D_5N.

Figure S21. ^{13}C-NMR spectrum of pacificusoside C (**3**) in C_5D_5N.

Figure S22. ^{1}H-^{1}H-COSY spectrum of pacificusoside C (**3**) in C_5D_5N.

Figure S23. HSQC spectrum of pacificusoside C (**3**) in C_5D_5N.

Figure S24. HMBC spectrum of pacificusoside C (**3**) in C_5D_5N.

Figure S25. ROESY spectrum of pacificusoside C (**3**) in C_5D_5N.

Figure S1. (–)HRESIMS spectrum of pacificusoside A (**1**).

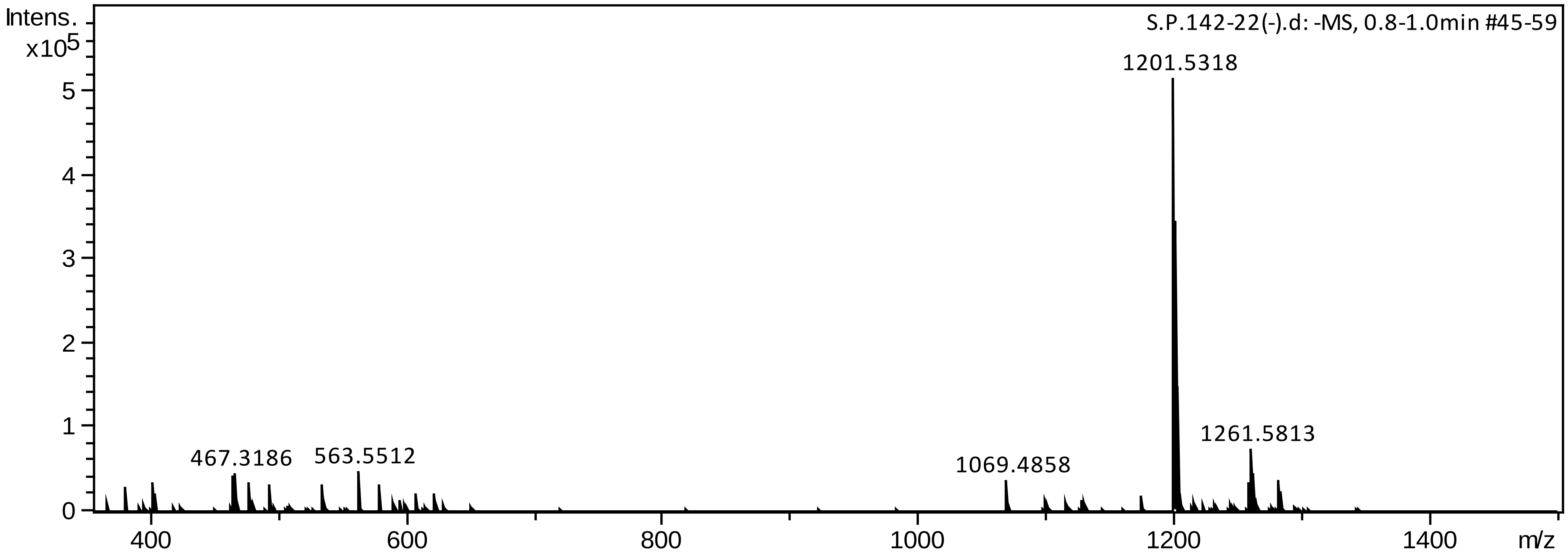

Figure S2. IR spectrum of pacificusoside A (**1**) in KBr.

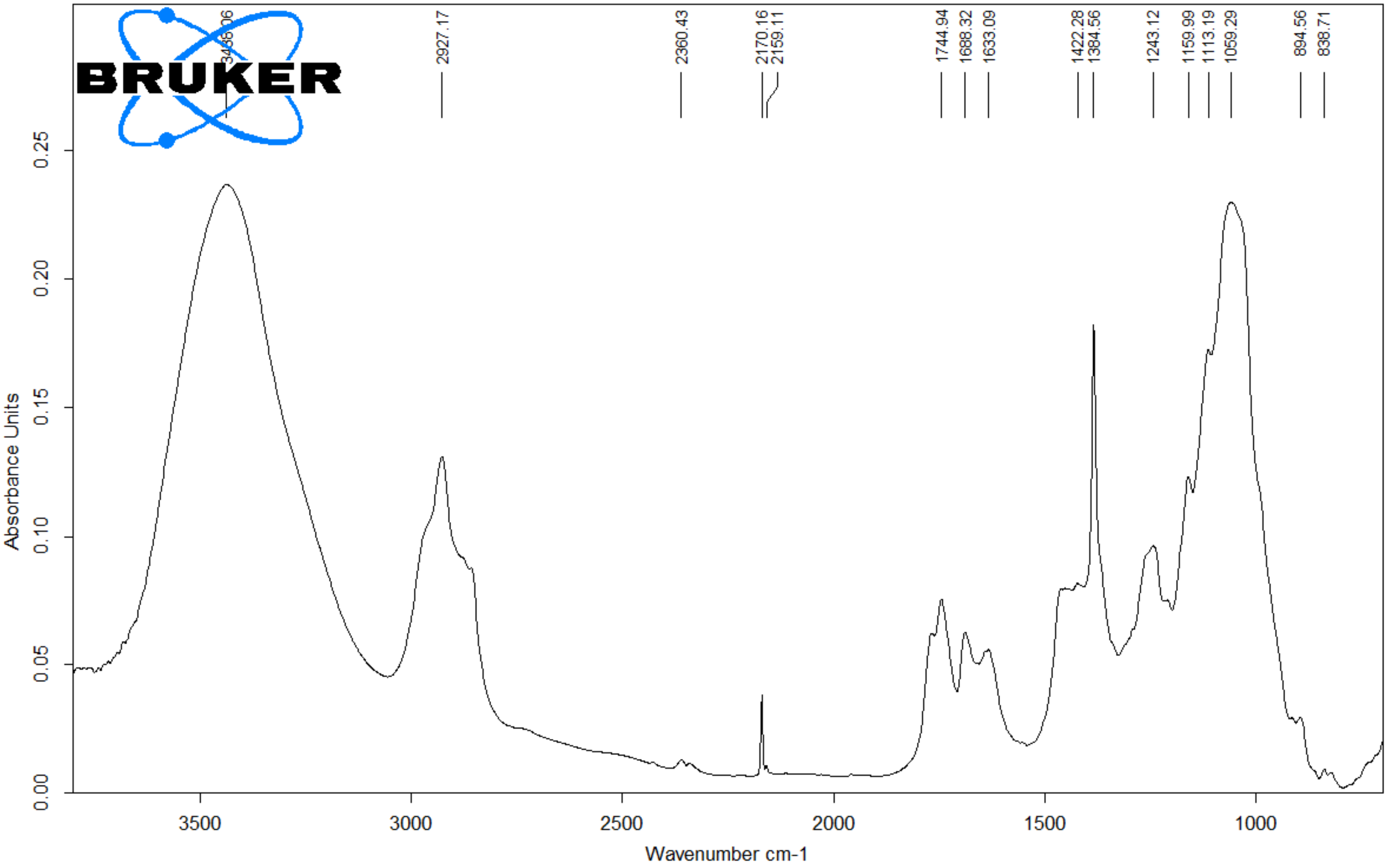

Figure S3. UV spectrum of pacificusoside A (**1**) in MeOH.

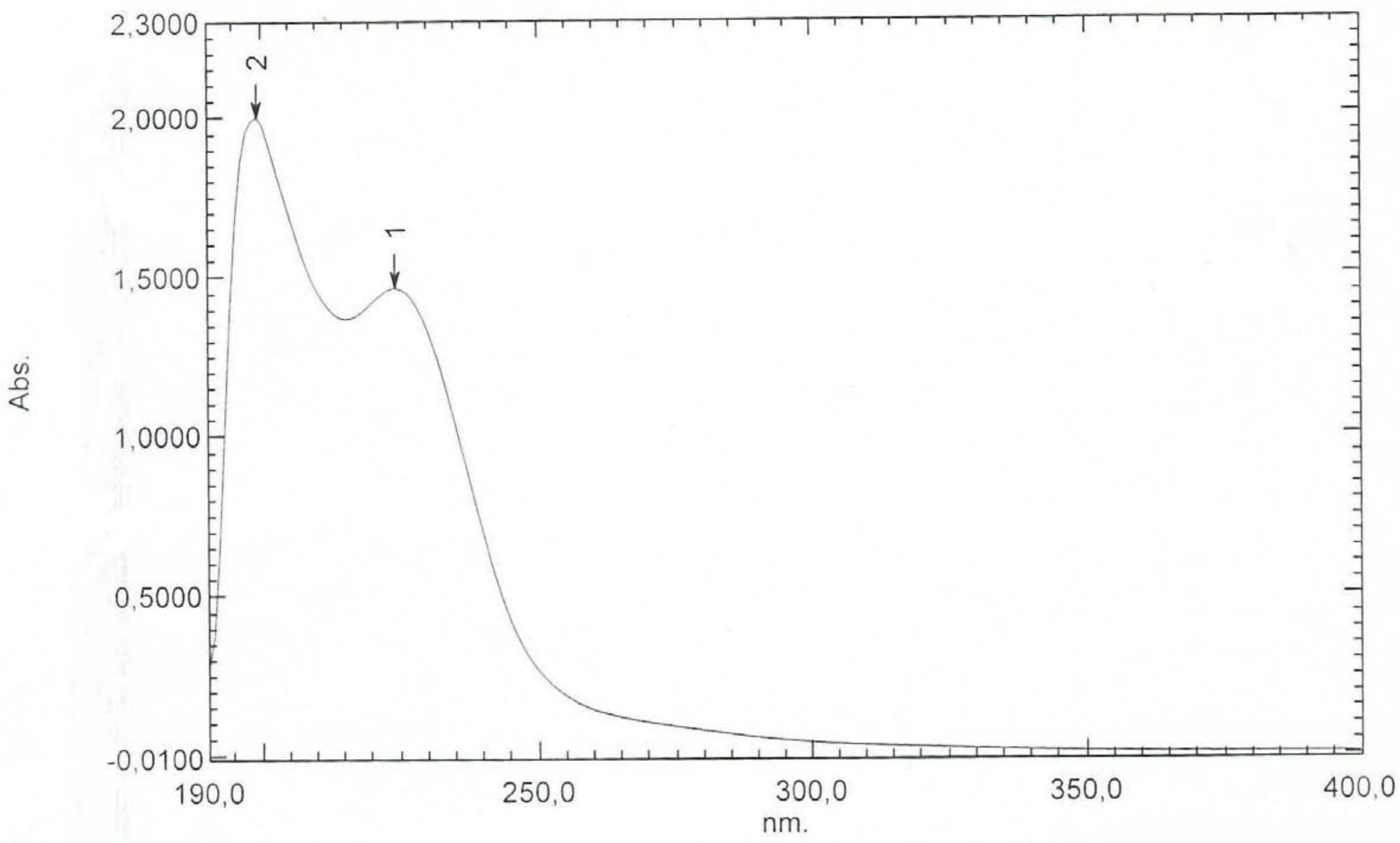

Figure S4. ^{1}H NMR spectrum of pacificusoside A (**1**) in C_5D_5N.

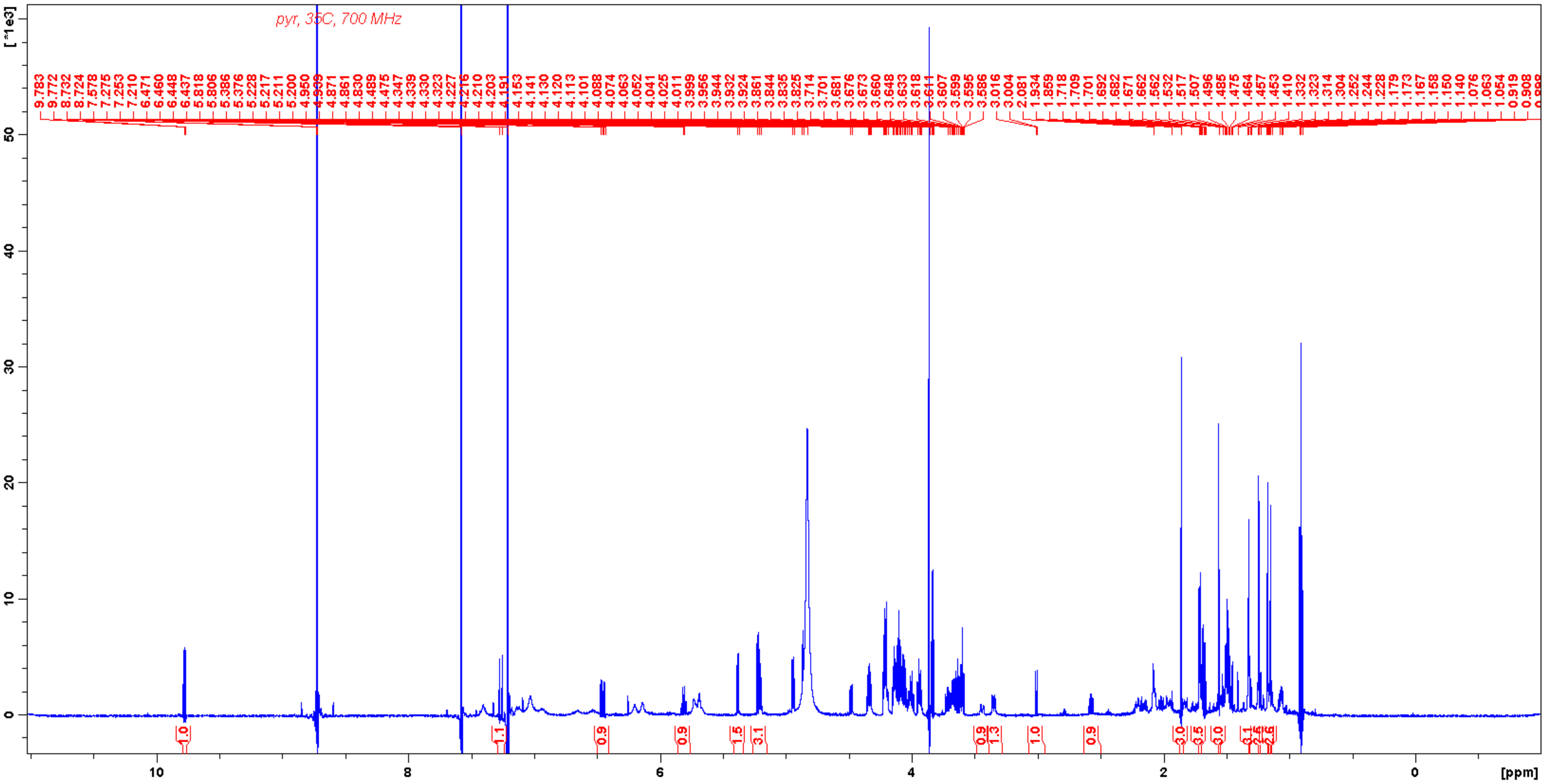

Figure S5. ^{13}C NMR spectrum of pacificusoside A (**1**) in C_5D_5N.

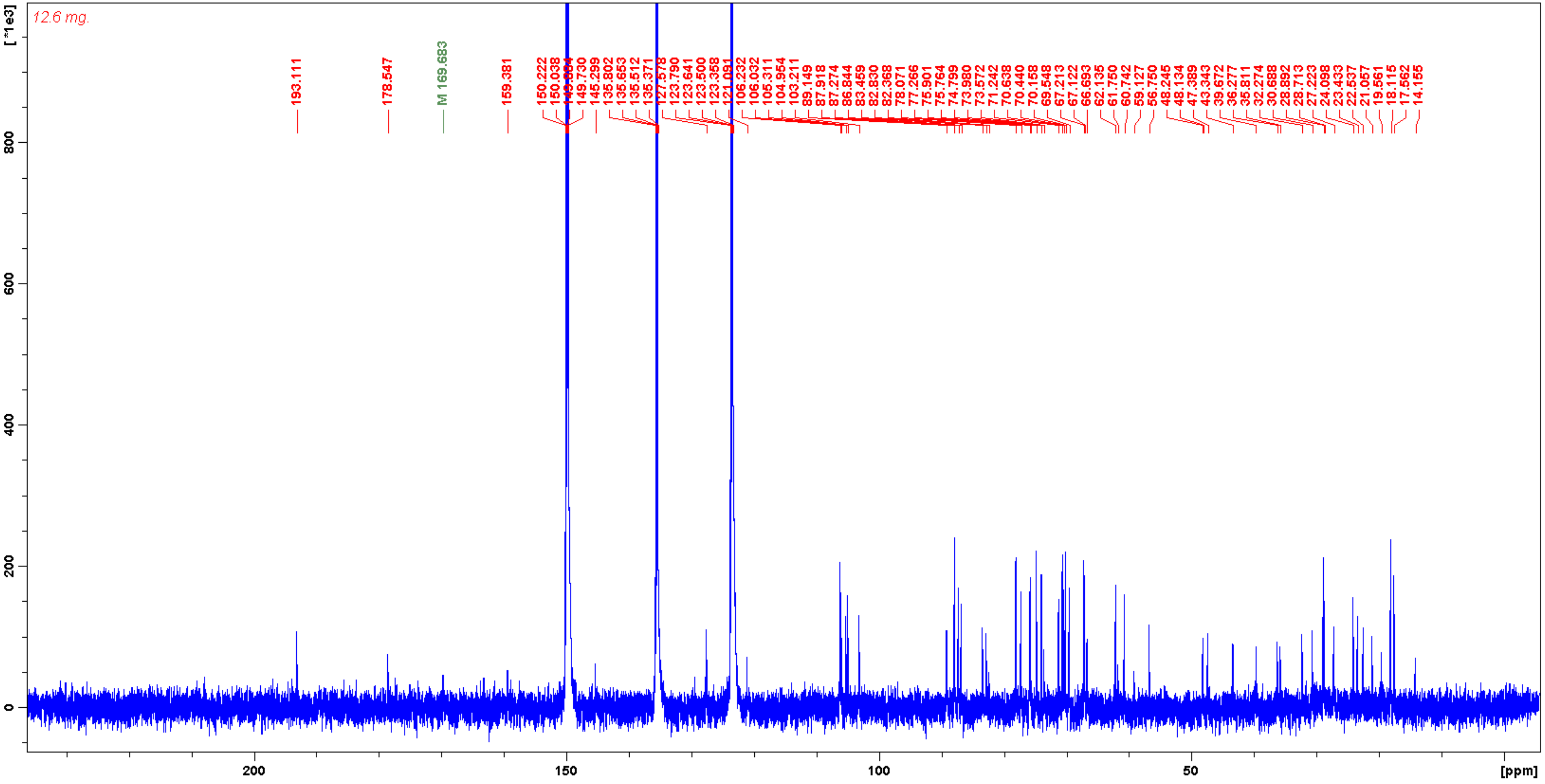

Figure S6. ^{1}H-^{1}H COSY spectrum of pacificusoside A (**1**) in C_5D_5N.

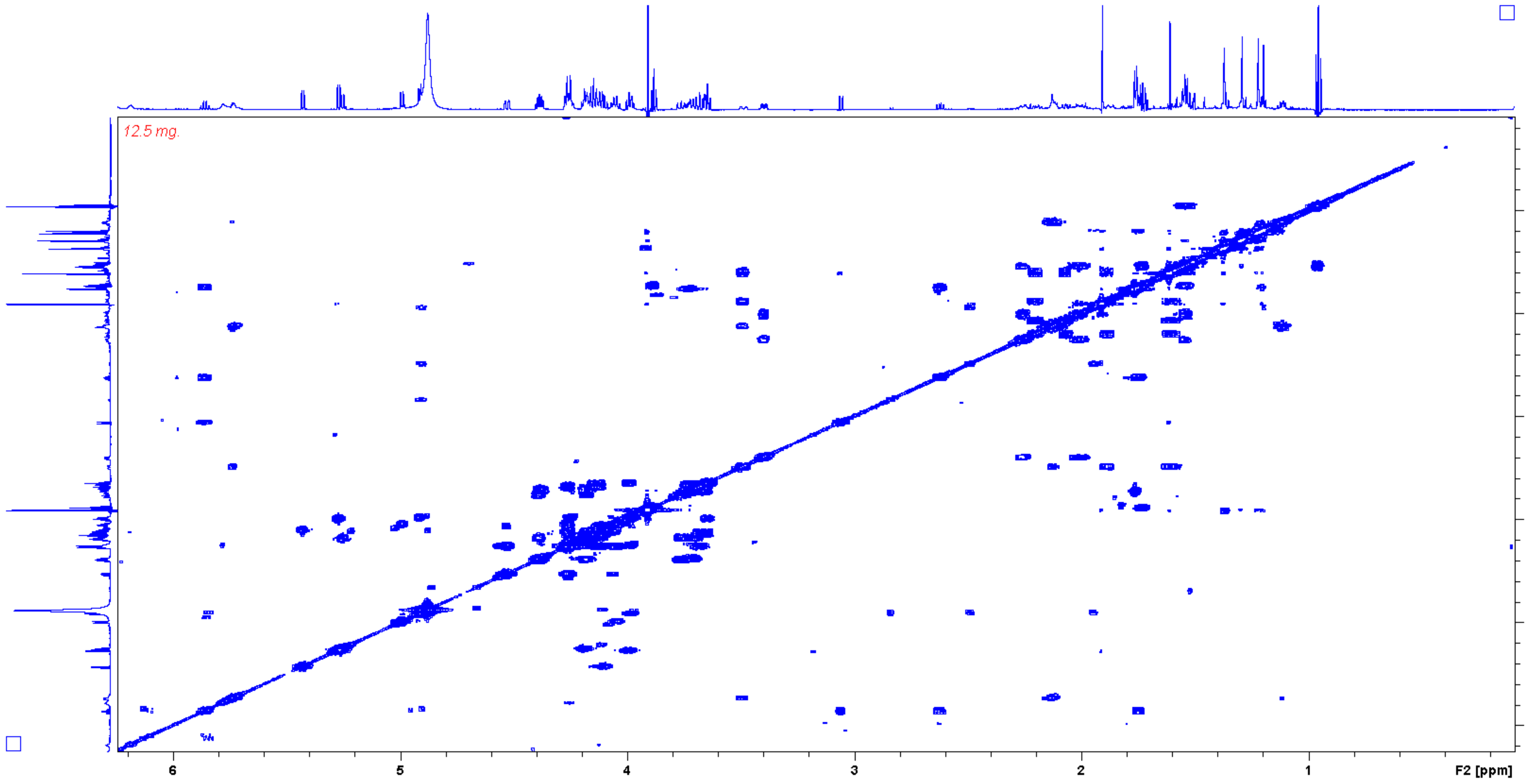

Figure S7. HSQC spectrum of pacificusoside A (**1**) in C_5D_5N.

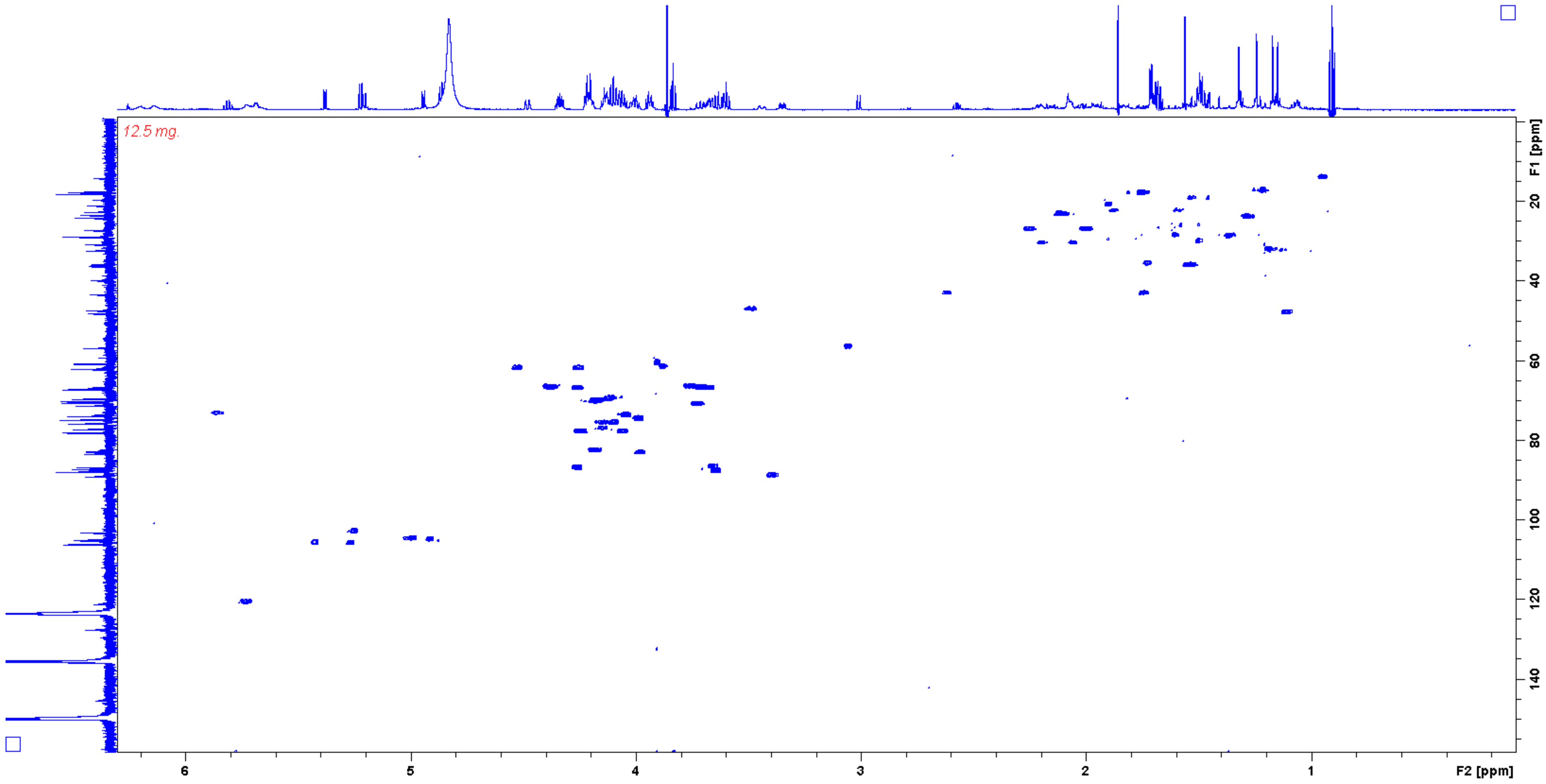

Figure S8. HMBC spectrum of pacificusoside A (**1**) in C_5D_5N.

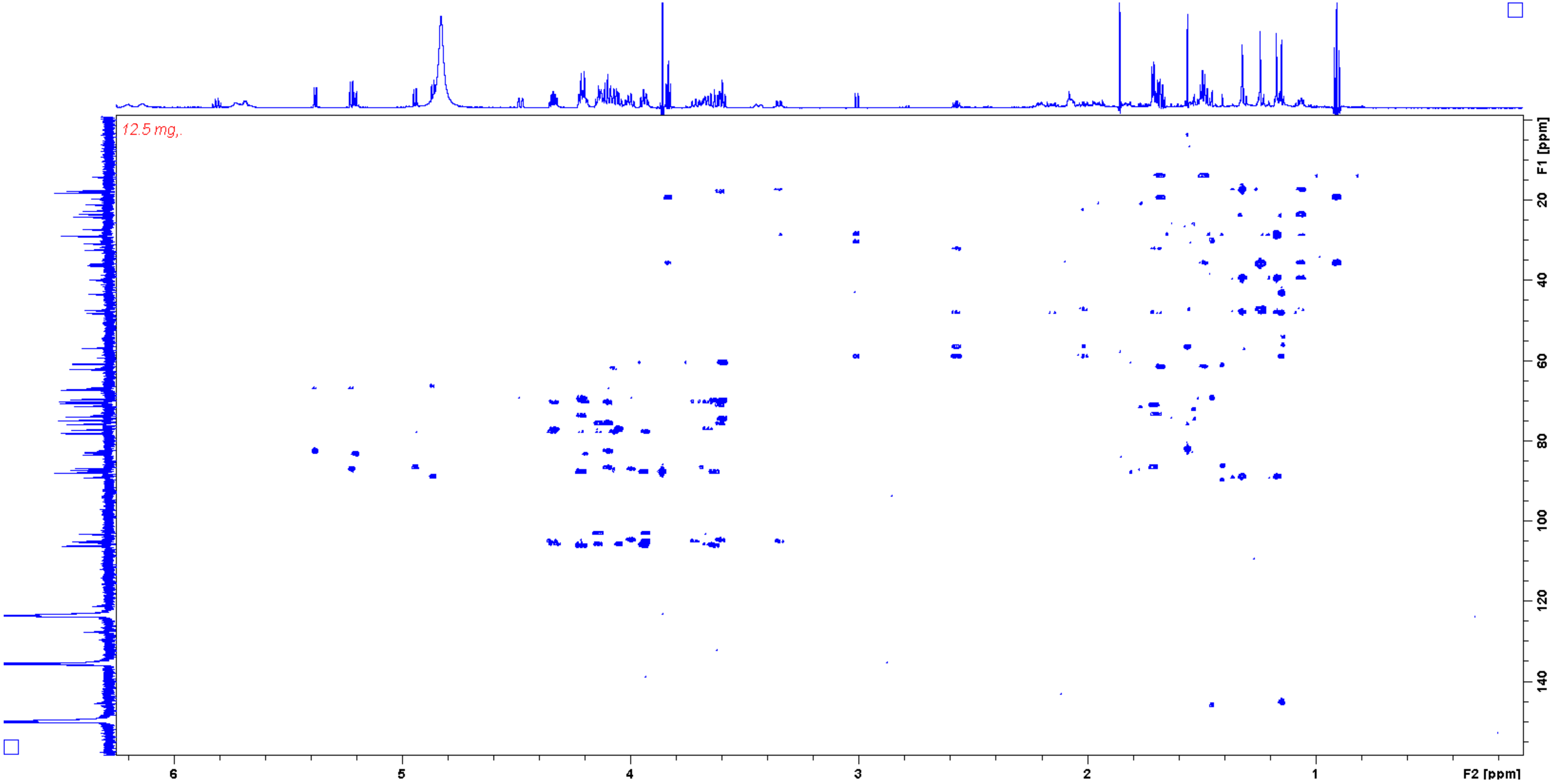

Figure S9. ROESY spectrum of pacificusoside A (**1**) in C_5D_5N.

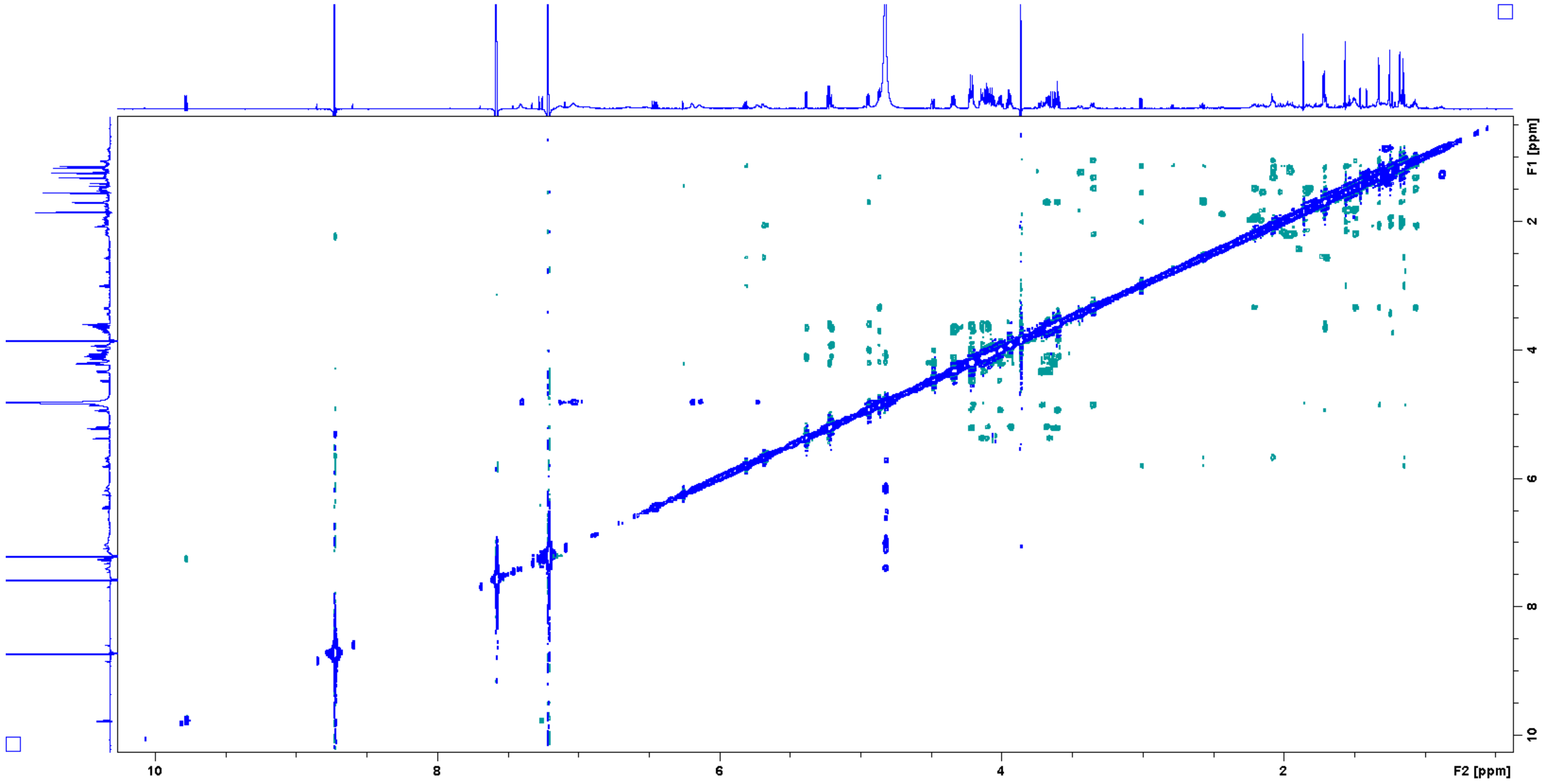

Figure S10. (–)HRESIMS spectrum of pacificusoside B (**2**).

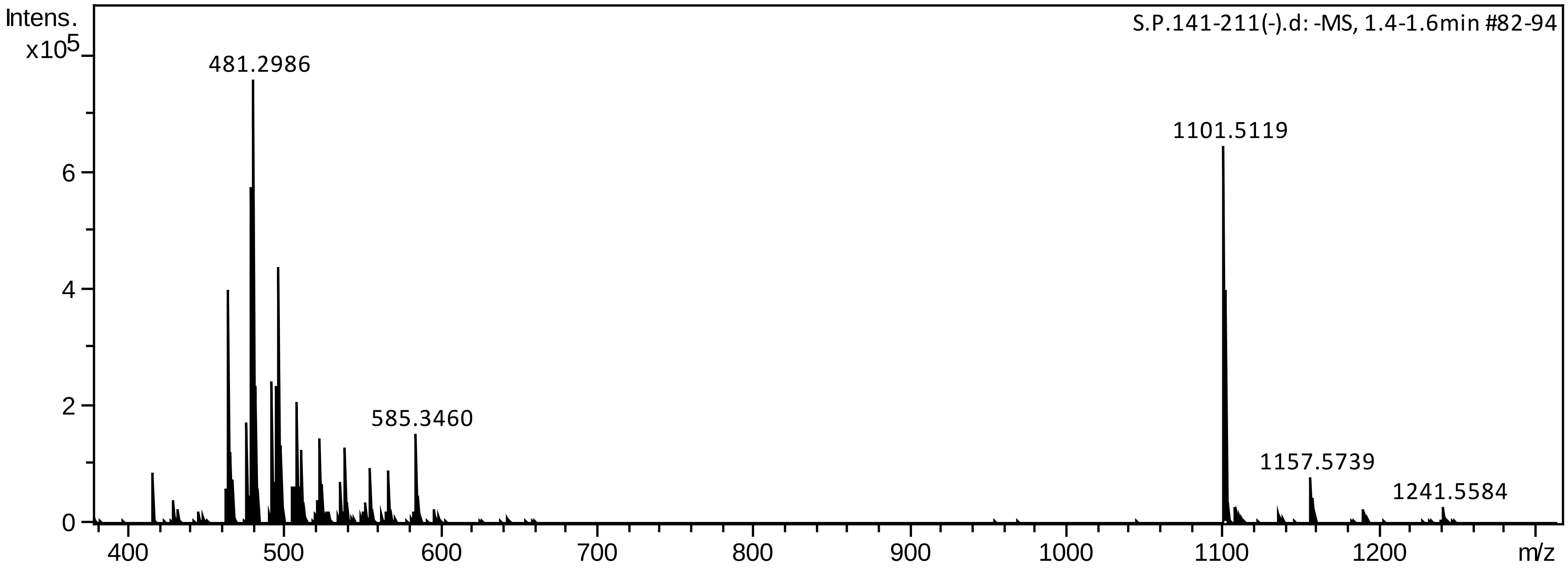

Figure S11. IR spectrum of pacificusoside B (**2**) in KBr.

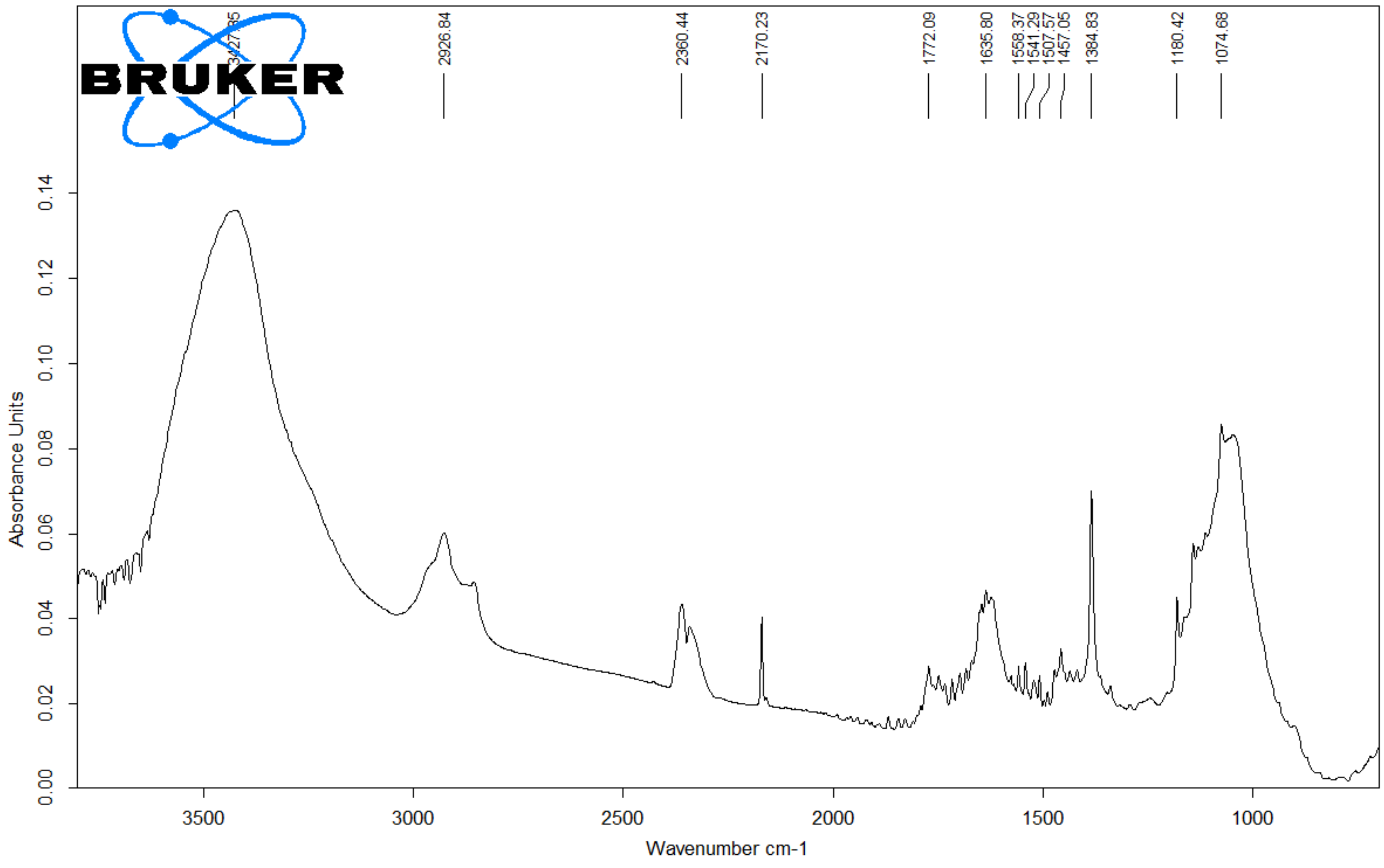

Figure S12. ^{1}H NMR spectrum of pacificusoside B (**2**) in C_5D_5N.

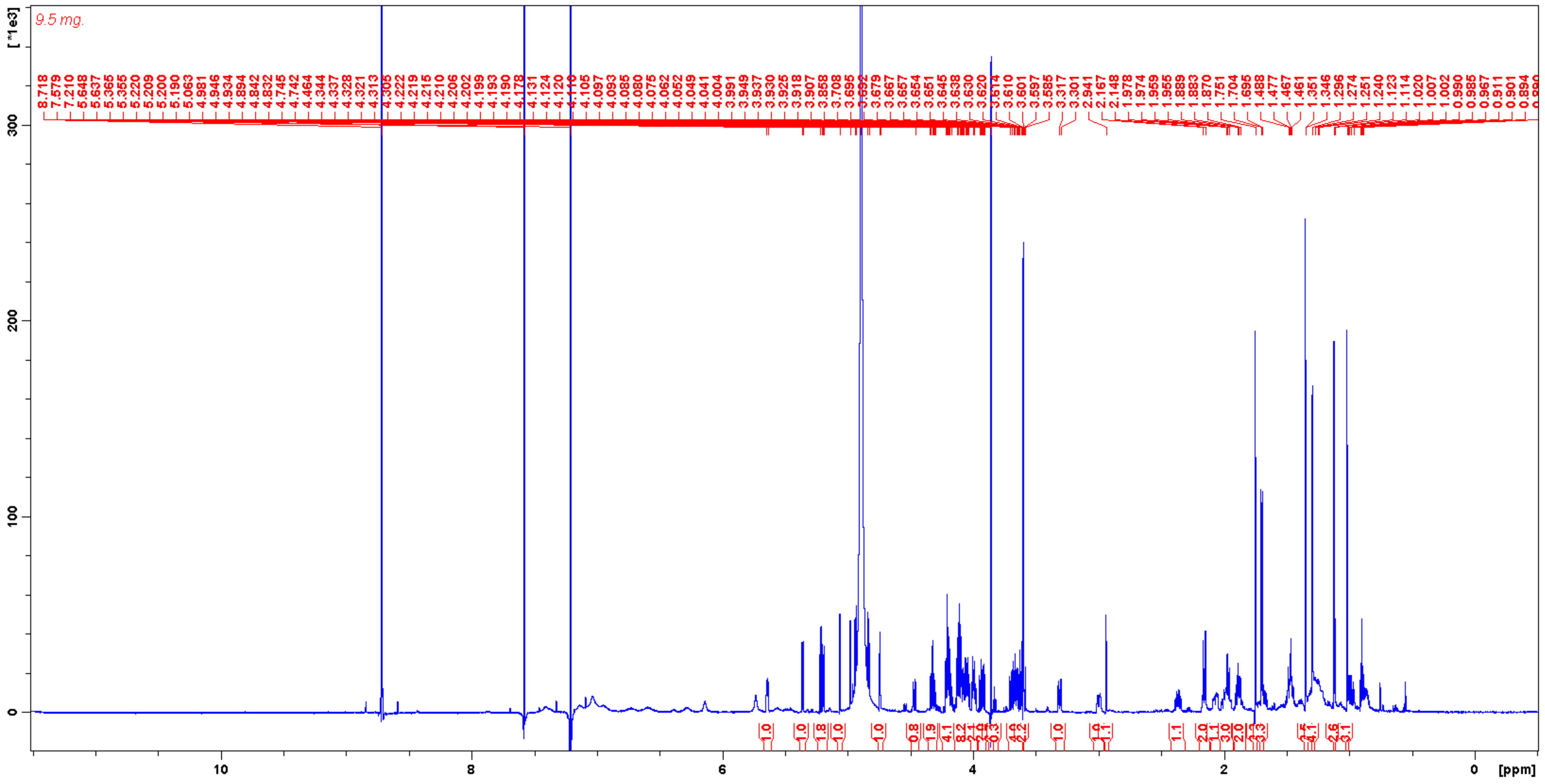

Figure S13. ^{13}C NMR spectrum of pacificusoside B (**2**) in C_5D_5N.

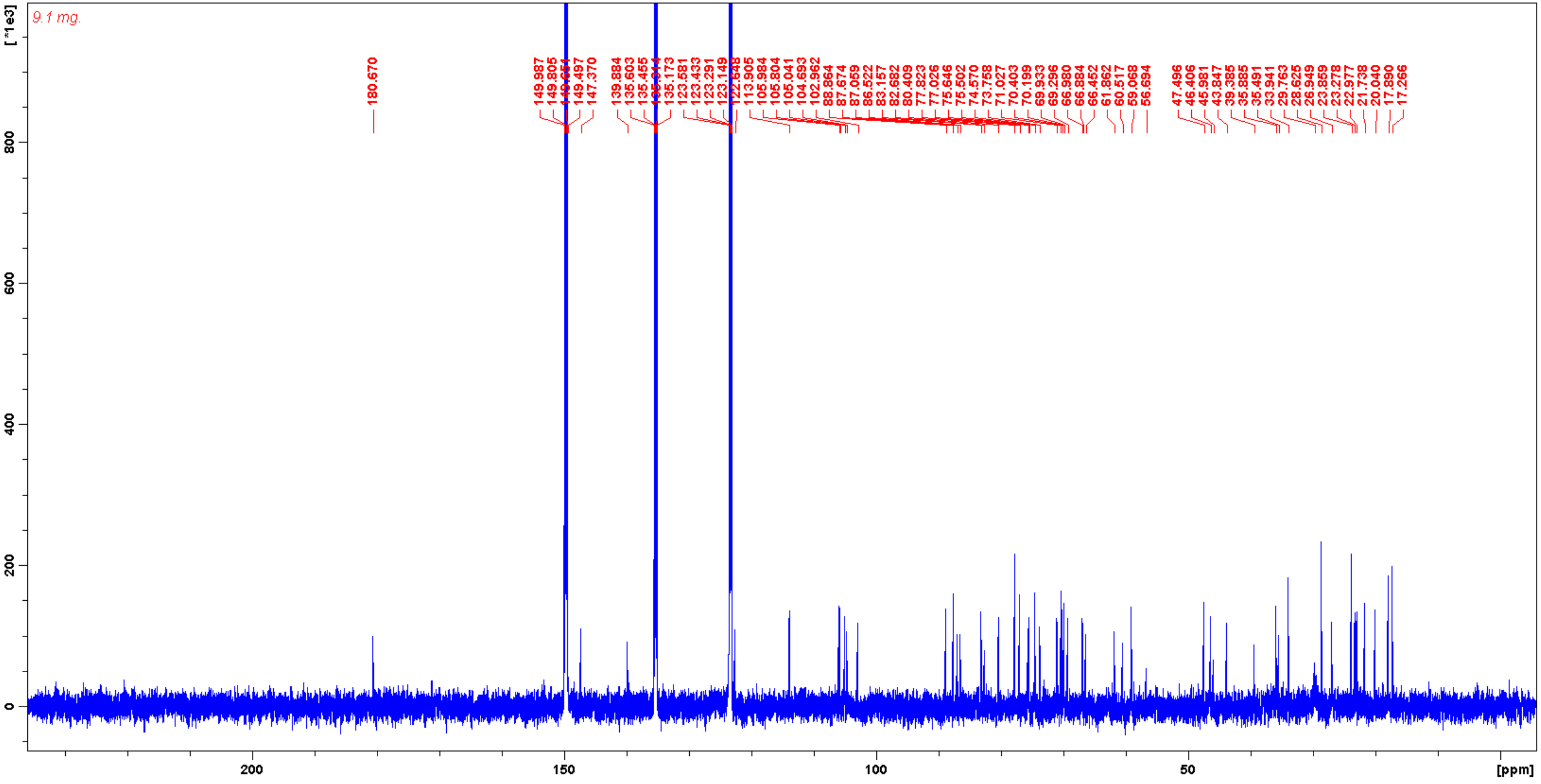

Figure S14. ^{1}H-^{1}H COSY spectrum of pacificusoside B (**2**) in C_5D_5N.

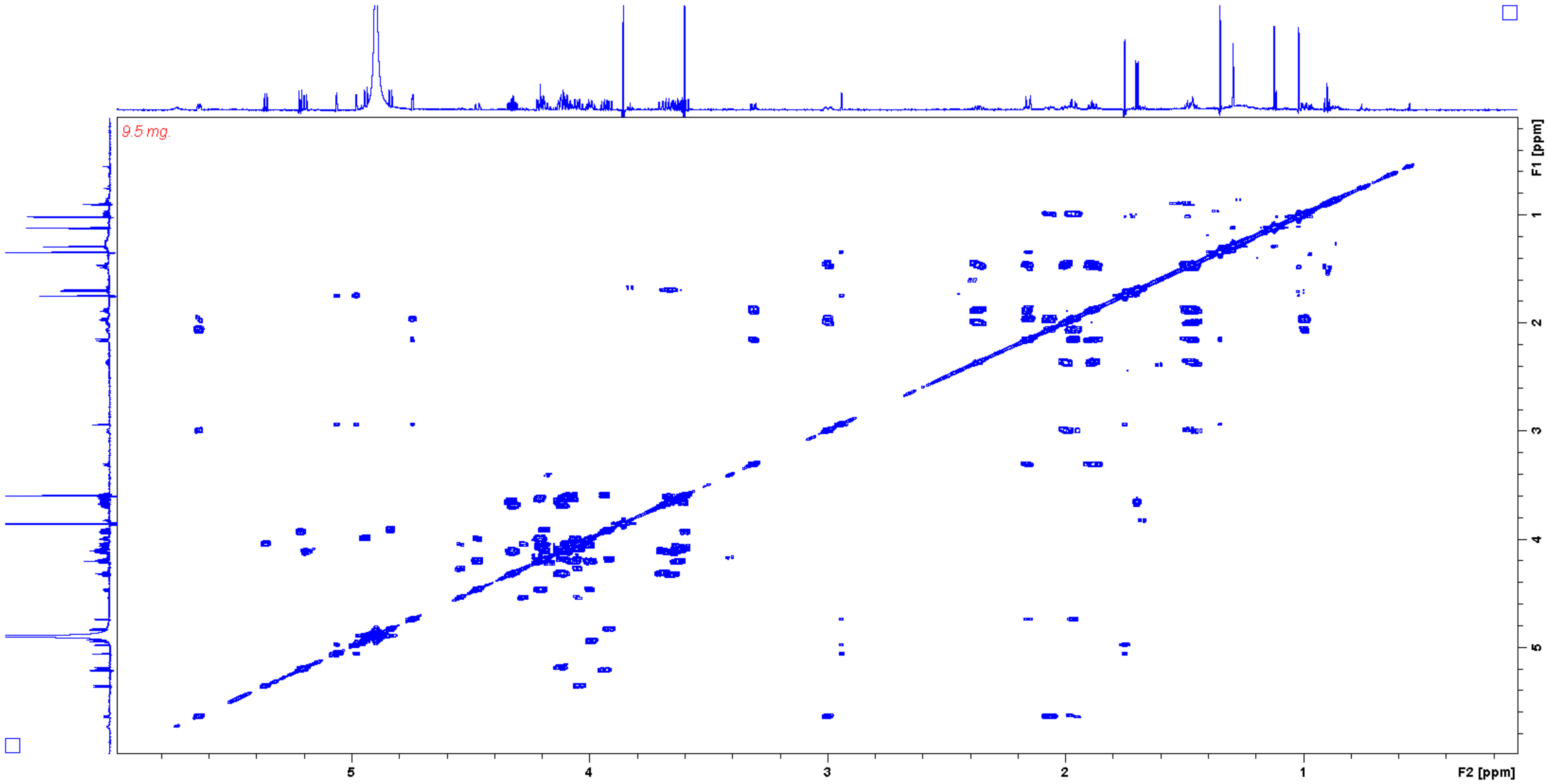

Figure S15. HSQC spectrum of pacificusoside B (**2**) in C_5D_5N.

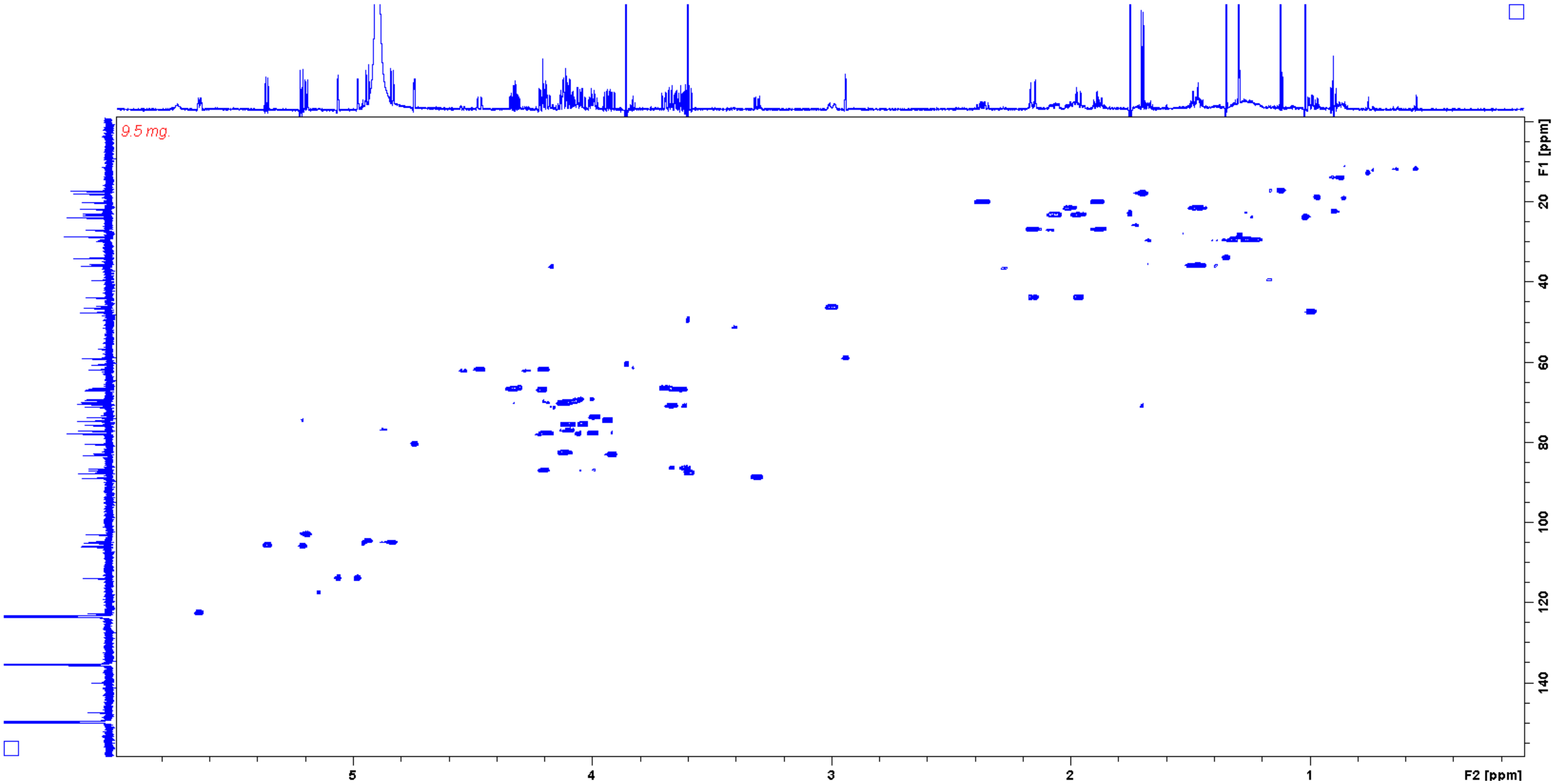

Figure S16. HMBC spectrum of pacificusoside B (**2**) in C_5D_5N.

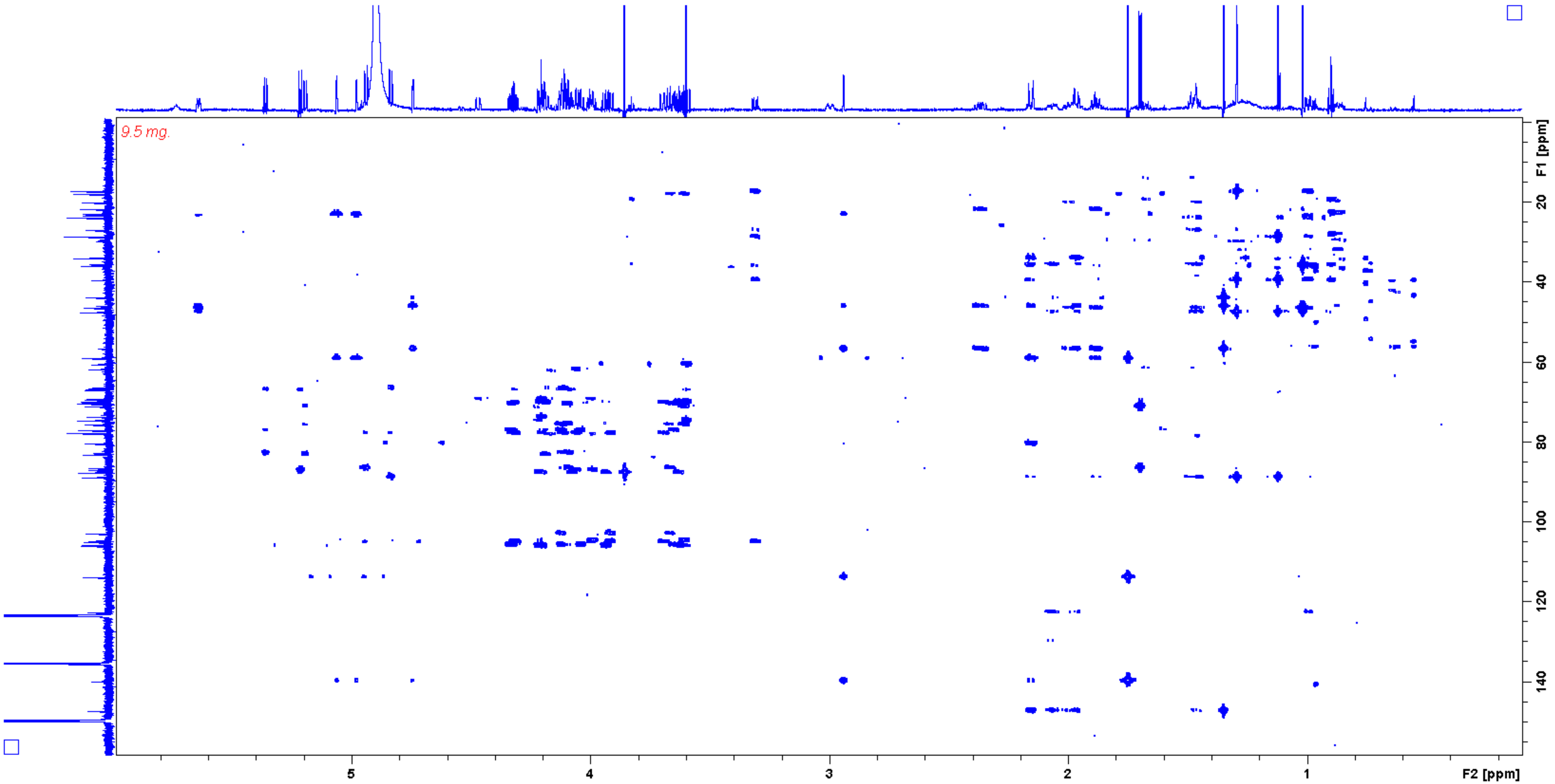

Figure S17. ROESY spectrum of pacificusoside B (**2**) in C_5D_5N.

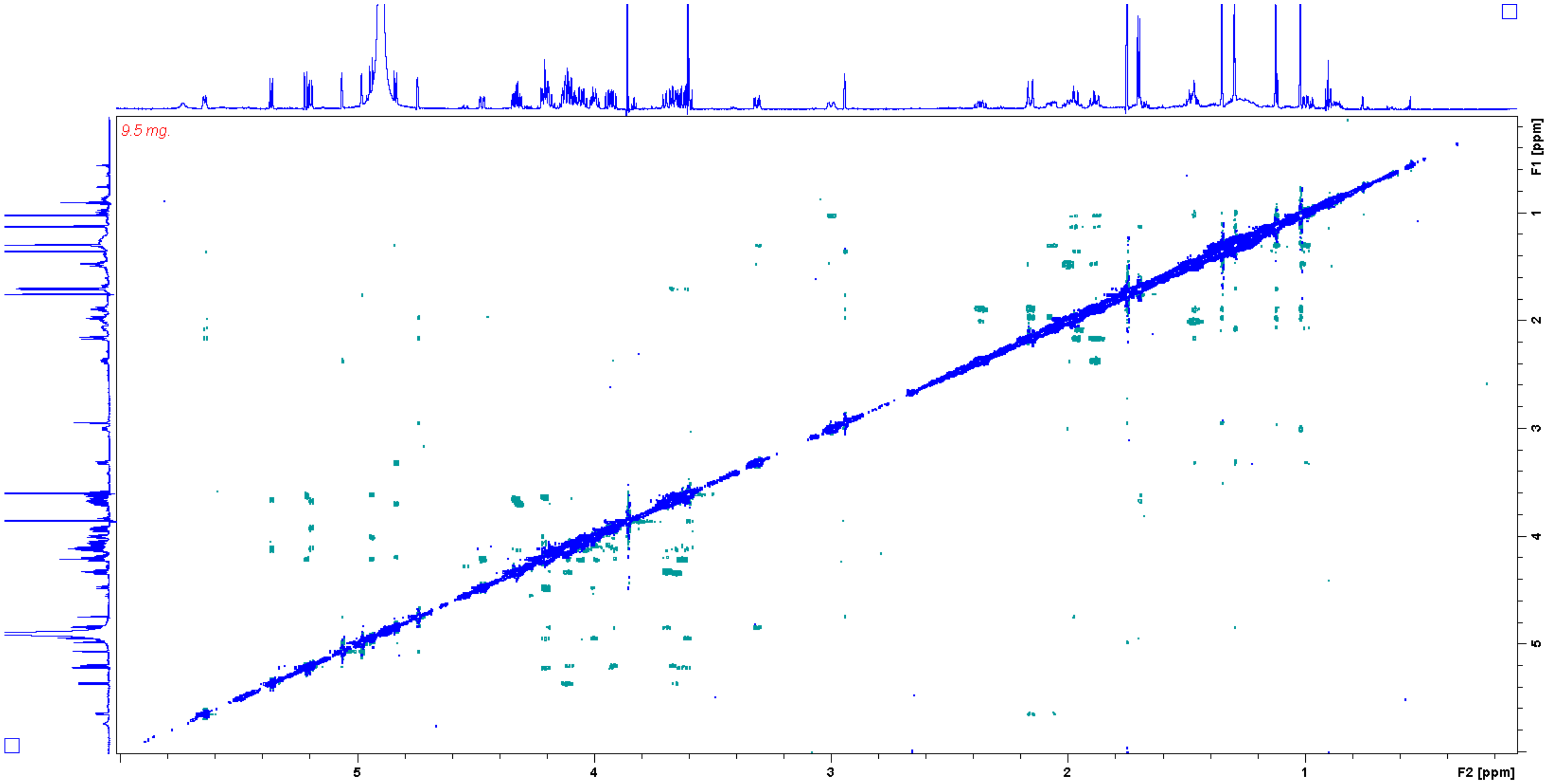

Figure S18. (–)HRESIMS spectrum of pacificusoside C (**3**).

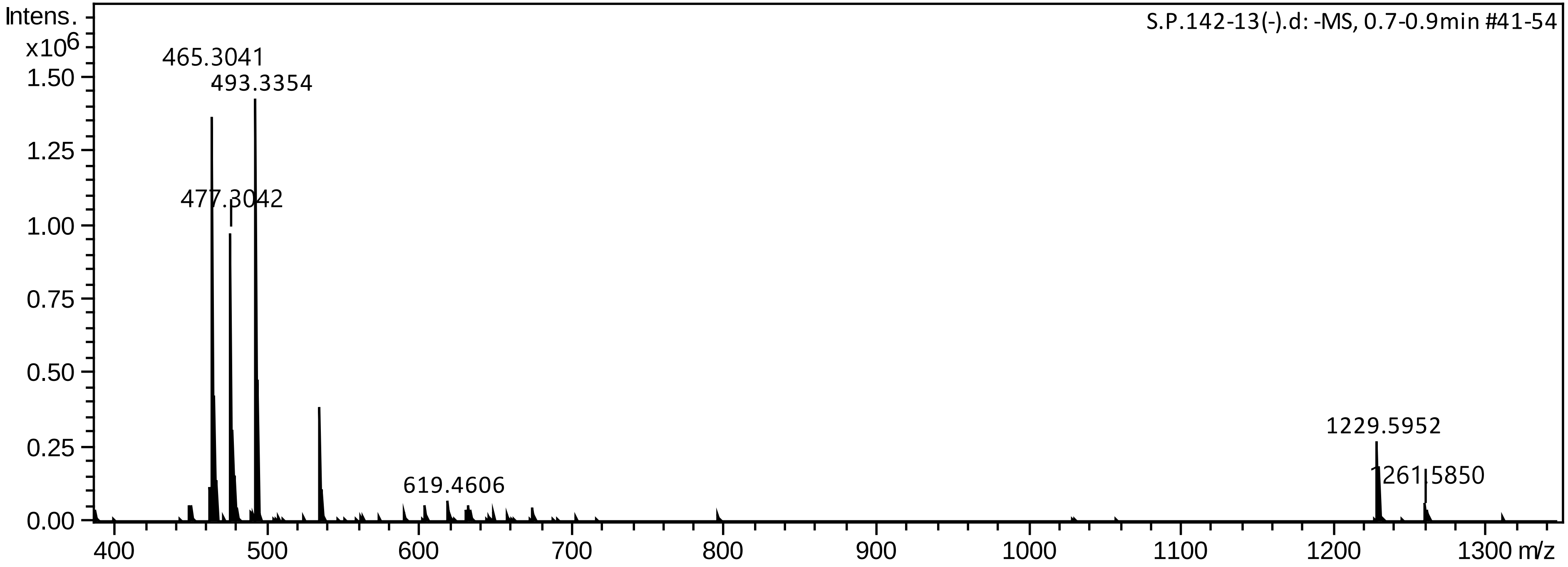

Figure S19. IR spectrum of pacificusoside C (**3**) in KBr.

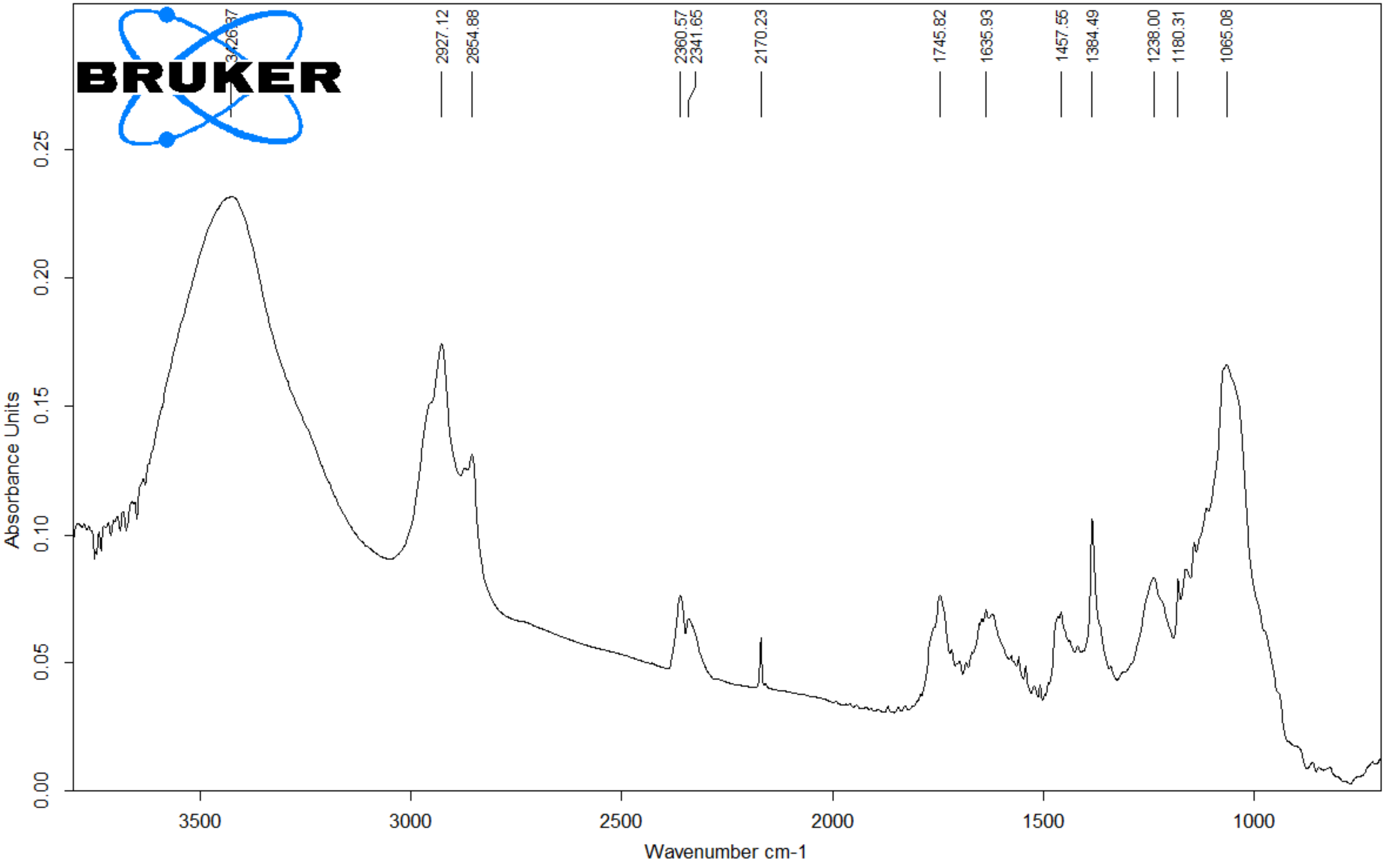

Figure S20. ^{1}H NMR spectrum of pacificusoside C (**3**) in C_5D_5N.

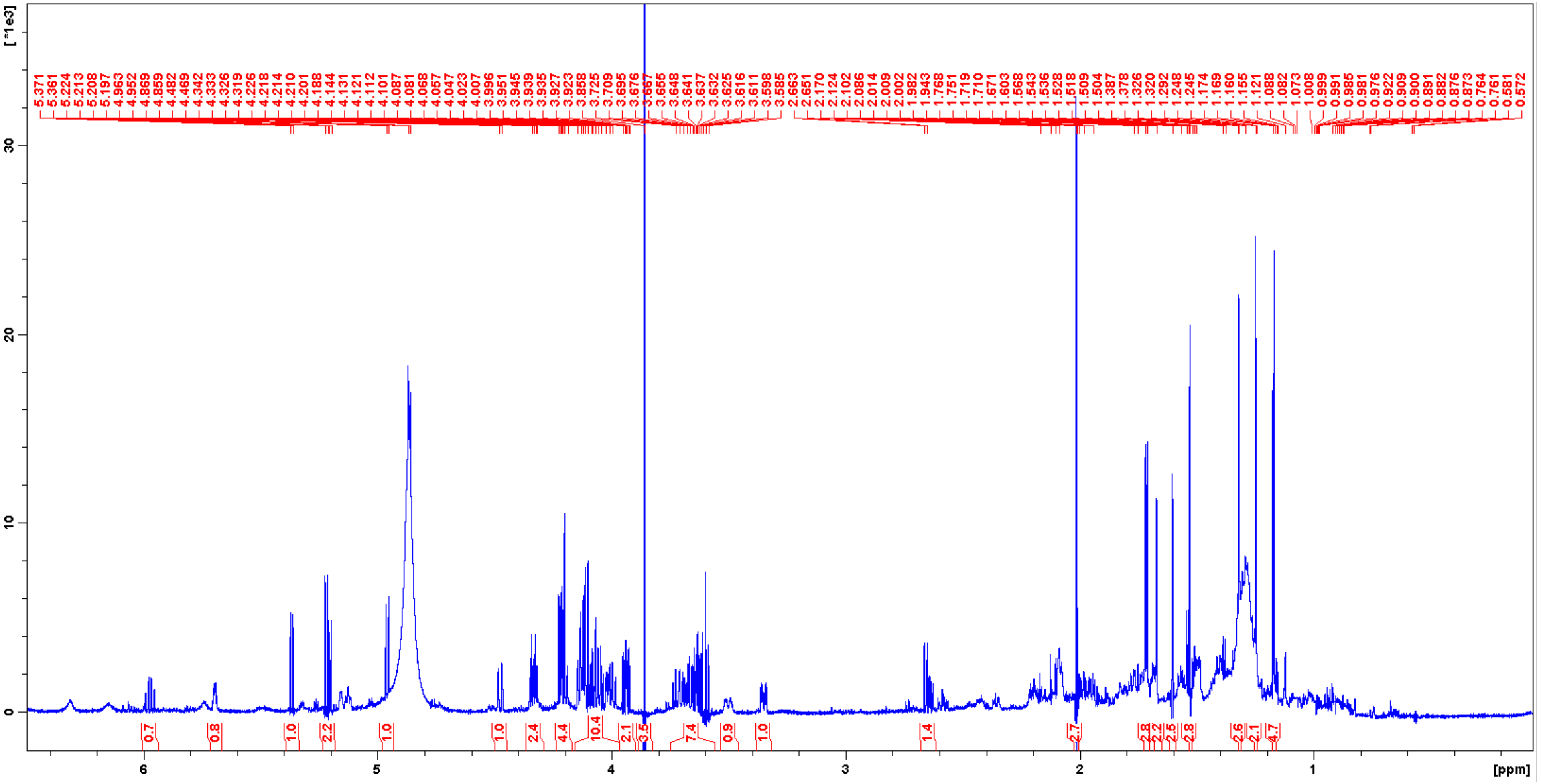

Figure S21. ^{13}C NMR spectrum of pacificusoside C (**3**) in C_5D_5N.

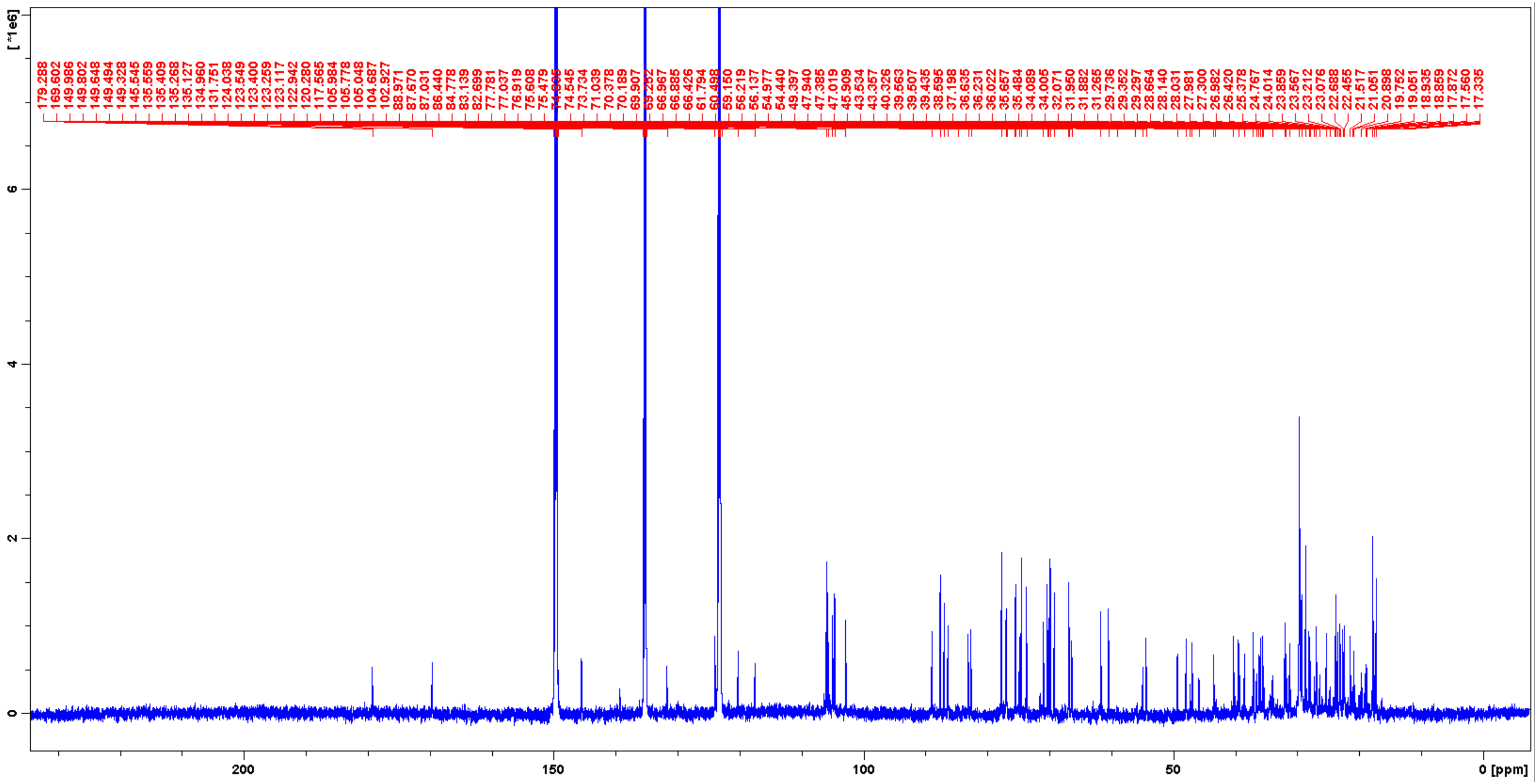

Figure S22. ^{1}H-^{1}H COSY spectrum of pacificusoside C (**3**) in C_5D_5N.

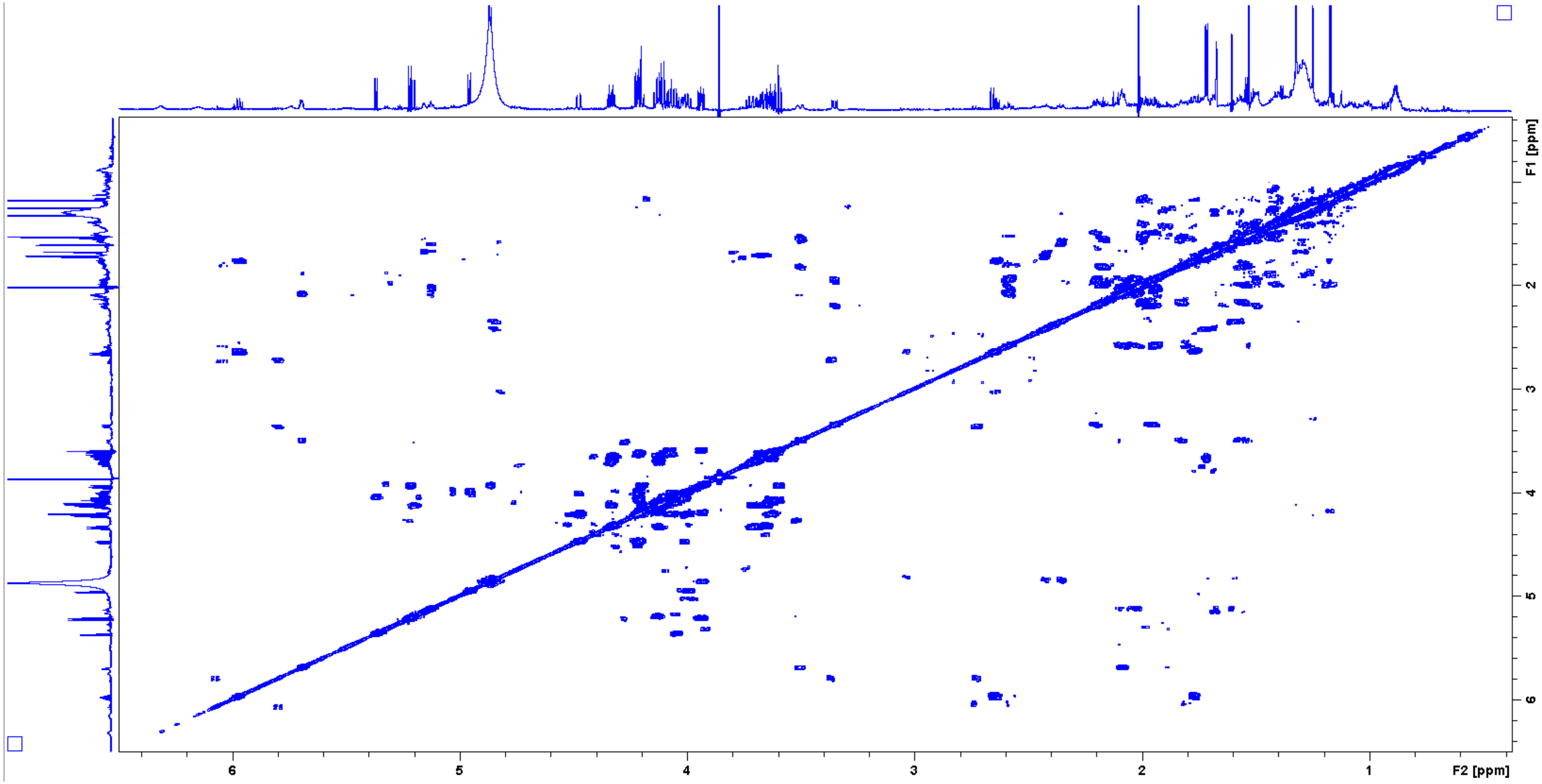

Figure S23. HSQC spectrum of pacificusoside C (**3**) in C_5D_5N.

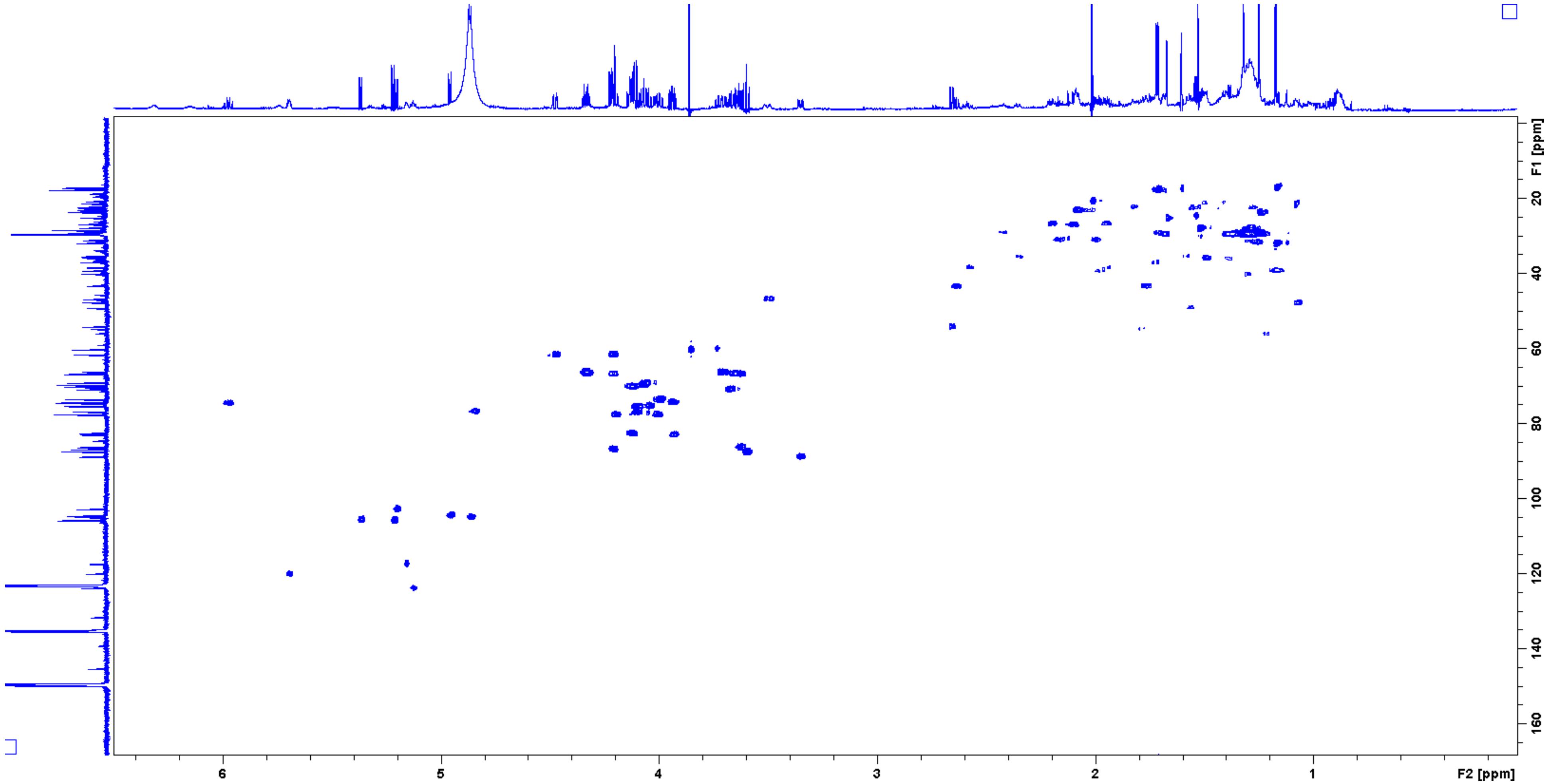

Figure S24. HMBC spectrum of pacificusoside C (**3**) in C_5D_5N.

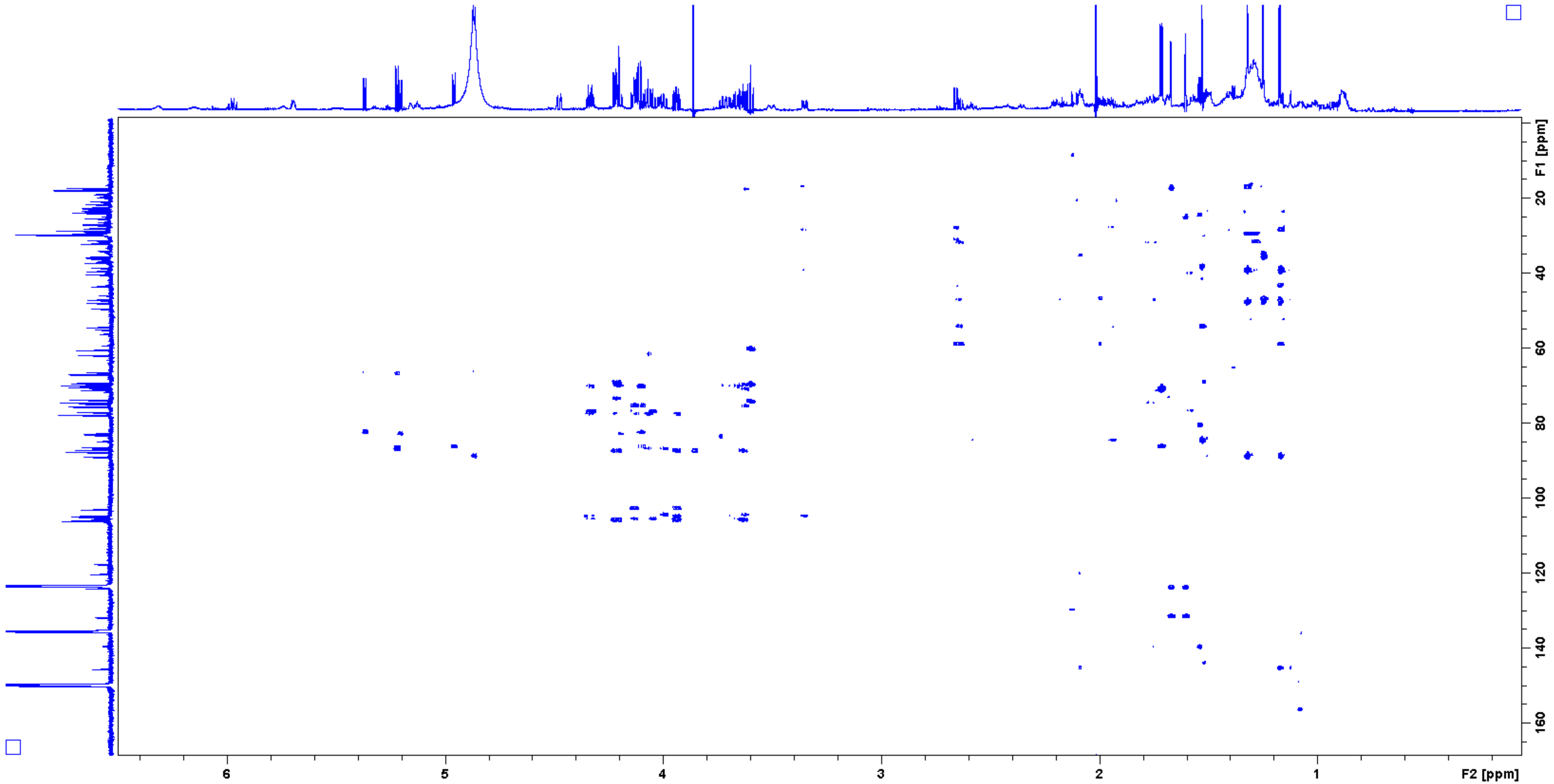

Figure S25. ROESY spectrum of pacificusoside C (**3**) in C_5D_5N.

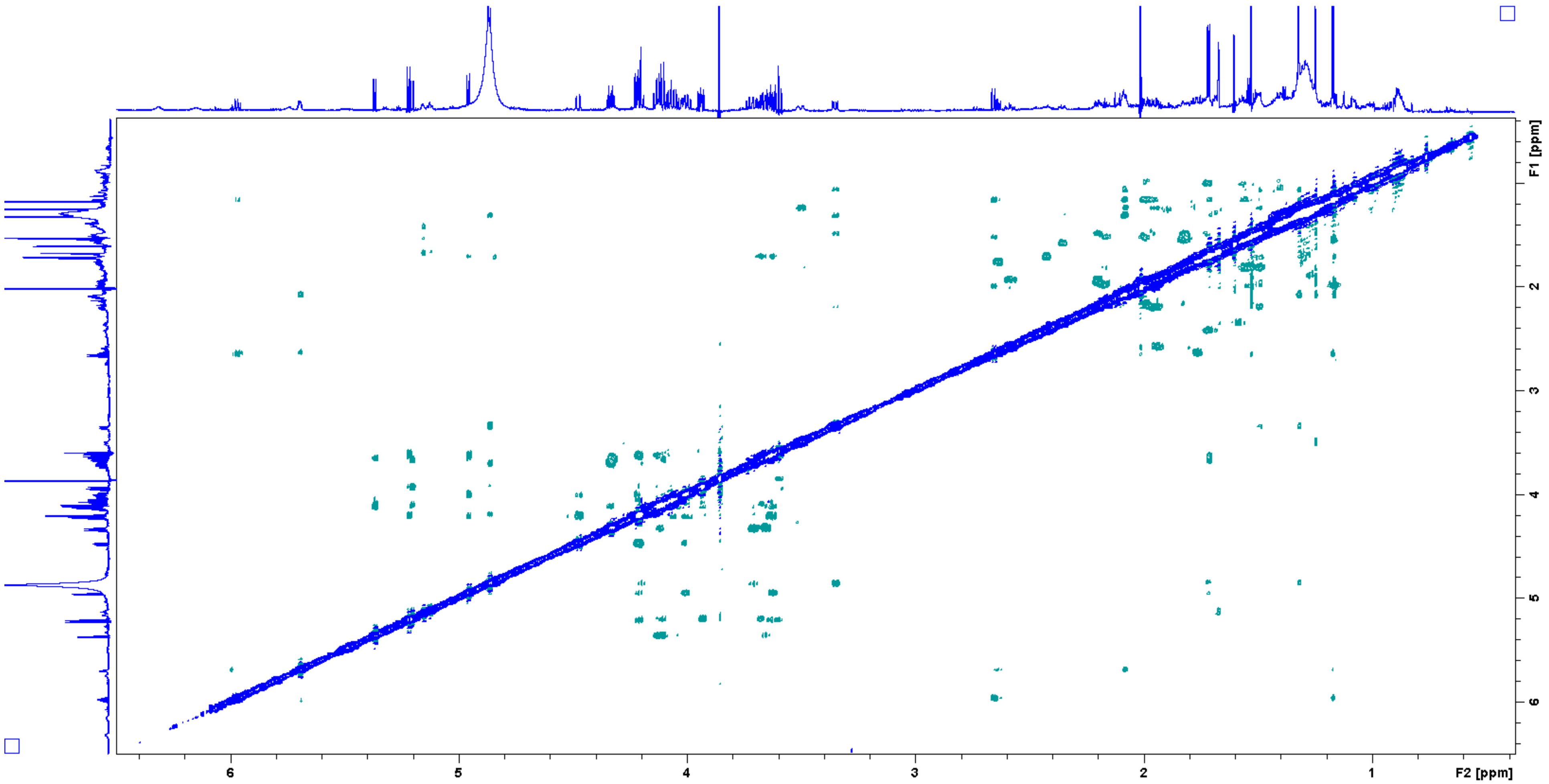

Supplement: Supplementary file 1 [file biomolecules-11-00427-s001.pdf]
